# A Systematic Review and Meta-Analysis of microRNA Profiling Studies in Chronic Kidney Diseases

**DOI:** 10.3390/ncrna10030030

**Published:** 2024-05-03

**Authors:** Gantsetseg Garmaa, Stefania Bunduc, Tamás Kói, Péter Hegyi, Dezső Csupor, Dariimaa Ganbat, Fanni Dembrovszky, Fanni Adél Meznerics, Ailar Nasirzadeh, Cristina Barbagallo, Gábor Kökény

**Affiliations:** 1 Institute of Translational Medicine, Semmelweis University, Nagyvárad tér 4, 1089 Budapest, Hungary; gantsetseg.garmaa@gmail.com (G.G.); ailarr.nz@gmail.com (A.N.); 2 Center for Translational Medicine, Semmelweis University, Üllői út 26, 1085 Budapest, Hungary; stfnbndc@gmail.com (S.B.); samatiok@gmail.com (T.K.); hegyi2009@gmail.com (P.H.); csupor.dezso@gmail.com (D.C.); dembrovszky.f@gmail.com (F.D.); meznerics.fanni@stud.semmelweis.hu (F.A.M.); 3 Department of Pathology, School of Medicine, Mongolian National University of Medical Sciences, Ulan-Bator 14210, Mongolia; dariimaa.ganbat21@gmail.com; 4 Faculty of Medicine, Carol Davila University of Medicine and Pharmacy, Dionisie Lupu Street 37, 020021 Bucharest, Romania; 5 Fundeni Clinical Institute, Fundeni Street 258, 022328 Bucharest, Romania; 6 Division of Pancreatic Diseases, Heart and Vascular Center, Semmelweis University, Baross út 22-24, 1085 Budapest, Hungary; 7 Department of Stochastics, Institute of Mathematics, Budapest University of Technology and Economics, Műegyetem rkp. 3, 1111 Budapest, Hungary; 8 Institute for Translational Medicine, Medical School, University of Pécs, 7624 Pécs, Hungary; 9 Institute of Clinical Pharmacy, University of Szeged, Szikra utca 8, 6725 Szeged, Hungary; 10 Department of Public Health, Graduate School of Medicine, International University of Health and Welfare, Tokyo 107-840, Japan; 11 Department of Dermatology, Venereology and Dermatooncology, Semmelweis University, Mária utca 41, 1085 Budapest, Hungary; 12 Section of Biology and Genetics “G. Sichel”, Department of Biomedical and Biotechnological Sciences, University of Catania, 95123 Catania, Italy; cbarbagallo@unict.it; 13 International Nephrology Research and Training Center, Semmelweis University, Nagyvárad tér 4, 1089 Budapest, Hungary

**Keywords:** chronic kidney disease, meta-analysis, microRNA, urine, blood

## Abstract

Chronic kidney disease (CKD) represents an increasing health burden. Evidence suggests the importance of miRNA in diagnosing CKD, yet the reports are inconsistent. This study aimed to determine novel miRNA biomarkers and potential therapeutic targets from hypothesis-free miRNA profiling studies in human and murine CKDs. Comprehensive literature searches were conducted on five databases. Subgroup analyses of kidney diseases, sample types, disease stages, and species were conducted. A total of 38 human and 12 murine eligible studies were analyzed using Robust Rank Aggregation (RRA) and vote-counting analyses. Gene set enrichment analyses of miRNA signatures in each kidney disease were conducted using DIANA-miRPath v4.0 and MIENTURNET. As a result, top target genes, Gene Ontology terms, the interaction network between miRNA and target genes, and molecular pathways in each kidney disease were identified. According to vote-counting analysis, 145 miRNAs were dysregulated in human kidney diseases, and 32 were dysregulated in murine CKD models. By RRA, miR-26a-5p was significantly reduced in the kidney tissue of Lupus nephritis (LN), while miR-107 was decreased in LN patients’ blood samples. In both species, epithelial-mesenchymal transition, Notch, mTOR signaling, apoptosis, G2/M checkpoint, and hypoxia were the most enriched pathways. These miRNA signatures and their target genes must be validated in large patient cohort studies.

## 1. Introduction

Chronic kidney disease (CKD) is a broad term encompassing all primary diseases resulting in structural or functional kidney abnormalities or both that last at least three months [1]. CKD affects 13.4% of the adult population worldwide [2], identified as one of the leading causes of death globally [3]. In 2017, 5% of the world’s population had early CKD (stages 1–2) [4]. Many CKD patients lack clinical symptoms at the onset. They are only diagnosed in late stages with elevated serum creatinine, decreased estimated glomerular filtration rate (eGFR), structural changes in ultrasound imaging, or abnormal urinalysis. These diagnostic tools, the gold standard renal function tests, are not optimal for detecting early injury or dysfunction [5] to allow for an immediate therapeutic intervention [6]. Since CKD is expected to become the fifth leading cause of mortality worldwide by 2040 [7], searching for sensitive diagnostic biomarkers and novel therapeutic strategies is critically important.

Many novel, high-throughput “omics” technologies have recently made it easier to interrogate hundreds of potential biomarkers in renal disease [8]. Among them, microRNAs (miRNAs) have emerged as new diagnostic biomarkers [9] and therapeutic targets [10] with robust stability in urine [11], plasma [12], and tissue [9]. MiRNAs are short (~22 nucleotides) non-coding ribonucleic acid (ncRNA) molecules that bind to target messenger RNAs (mRNAs), resulting in their degradation and, therefore, translational repression. Most miRNAs can target multiple genes, which may lead to the suppression or activation of several hundred proteins at the same time for distinctive pathological or physiological processes. As a result, changes in their expression levels are frequently difficult to interpret.

The techniques for miRNA expression analyses are continuously developing; profiling studies, however, showed inconsistent results due to the variability in technological platforms (gene sequencing, microarray, and RT-qPCR), biological sample types, and study sample sizes. RT-qPCR is a reliable method for analyzing miRNA expressions. However, it is unsuitable for the high-throughput and hypothesis-free exploration of novel miRNAs that may be critical in kidney disease. Investigator bias can also influence the results.

Studies have identified dysregulation of miRNA in various types of kidney diseases in mice [10,13] and humans [14,15,16,17], suggesting their use as potential biomarkers for diagnosis [18] and as nucleic acid therapy [19]. Therefore, it is crucial to understand the miRNA expression patterns in various sample types during specific disease stages and their functional role in the development and progression of CKD. This information can be synthesized from published studies to gain valuable insights into the application of miRNAs as diagnostic, prognostic biomarkers, or even therapeutic agents. In addition, systematic reviews and meta-analyses comparing miRNA expression in different human biological samples in kidney diseases and translational comparisons of murine and human kidney diseases are scarce.

Several cohort studies regarding miRNA and CKDs have been published in peer-reviewed journals. However, the various techniques used to determine miRNAs made it difficult to conclude the importance of miRNA dysregulation during CKD. Recently, novel statistical methods, Robust Rank Aggregation (RRA) and vote-counting, implemented by researchers have provided a possibility to integrate miRNA lists from different platforms, such as gene sequencing, microarray, and RT-qPCR, without being affected by cut-off and normalization methods and complete raw data [20,21]. These methods have been used in previous meta-analyses in diabetic nephropathy, lupus nephritis, renal fibrosis [13,15,17], and heart failure [22].

This study aimed to determine the most dysregulated miRNAs in various kidney diseases, biological samples, and stages of CKD, only including studies with a healthy control group. In addition, miRNA signatures of the murine model of CKD were compared with possible overlaps in human CKD. Finally, gene set enrichment analysis of miRNA signatures was conducted to investigate target genes, Gene Ontology (GO) terms, interaction networks between miRNA and target genes, and molecular pathways for each disease.

## 2. Results

### 2.1. Search and Selection

**Figure 1:**
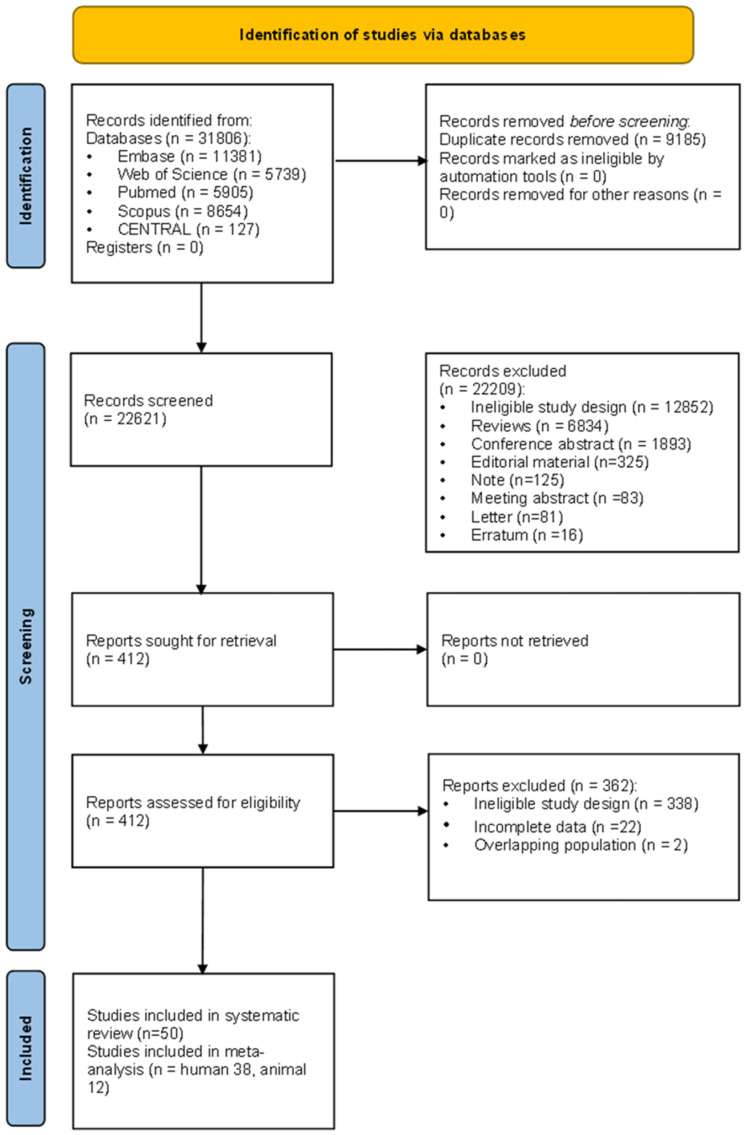
The flow of study selection.

Our search identified 412 records that mentioned miRNA and kidney disease, of which 50 miRNA expression profiling studies fulfilled the eligibility criteria. Among them, 38 were studies on CKD patients and healthy controls and 12 were conducted in murine experimental CKD models and sham controls. Besides the 50 studies, 10 human and 12 murine profiling studies were excluded from the selection process because ranked data were not available publicly or in response to a request from the corresponding author (detailed in Supplementary Methods). The study selection flow is detailed in Figure 1.

### 2.2. Study and Participant Characteristics

#### 2.2.1. Human Studies

The identified studies were conducted in 13 countries. A total of 762 CKD patients vs. 671 healthy controls (male 55.7% and female 44.3%) were included in our meta-analysis. The characteristics of eligible studies, participants, and miRNA profiling assays are shown in Table 1. Some studies specified patients who received routine medical treatment at the time of sample collection [23,24,25,26,27,28,29]. Only two studies included patients taking immunosuppressive treatment [26,30]. The studies used various platforms for determining miRNAs’ differential expression: microarray (n = 15), RNA sequencing (n = 17), PCR (n = 3), and NanoString (n = 3). The average number of tested miRNAs was 1406 (168 to 2588).

Disease types and biological samples included: Some eligible studies reported on more than one type of kidney disease or biological sample, which resulted in 55 individual results (further stated as “individual results”) retrieved from 38 articles. In detail, ten articles reported two to three different disease types separately [25,26,30,33,34,36,38,39,43,53]. Of the total 55 individual results, 13 focused solely on diabetic nephropathy (DN) [24,31,32,33,34,35,36,37,38,39,40,41,42], 10 focused on IgA nephropathy (IgAN) [34,39,43,44,45,46,47,48,49,54], and 4 did not specify the form of CKD [23,52,53,55]. The rest of the studies included lupus nephritis (LN) or post-streptococcal glomerulonephritis (PSGN) (n = 7) [26,27,28,29,33,51,56], focal segmental glomerulosclerosis (FSGS) (n = 5) [25,30,38,39,57] or minimal change disease (MCD) (n = 5) [25,30,34,38,43], membranous nephropathy (MN) (n = 5) [30,36,43,58,59], crescentic glomerulonephritis (n = 1) [34], membranoproliferative GN (MPGN) (n = 2) [39,50], and membranous glomerulonephropathy (MGN) (n = 1) [60].

A total of 21 studies reported on 645 urine samples (exosome and sediments), 18 studies reported on 513 blood samples (serum, plasma, plasma or serum exosome, and peripheral blood mononuclear lymphocytes (PBMCs)), and 15 studies reported on 301 kidney biopsy samples.

A total of 16 studies (reporting 18 individual results) recruited 257 early-stage CKD patients and 246 controls, and 36 studies (reporting 37 individual results) enrolled 530 late-stage CKD patients and 437 controls. The eGFR in early-stage CKD patients was >60 mL/min per 1.73 m^2^ (range: 60 to 160.3) and <60 mL/min per 1.73 m^2^ (range: 13.75 to 59) in the late stage.

#### 2.2.2. Murine Studies

We included experimental models of CKD with 70 diseased mice and 66 sham controls in the analysis. The characteristics of eligible studies, subjects, and miRNA profiling assays are shown in Table 2. Ishii [61] and colleagues reported two murine models of CKD. Therefore, we could retrieve 13 individual results from 12 articles. The average number of tested miRNAs was 1041 (ranging from 375 to 1916). In murine experimental models of CKD, there were seven diabetic nephropathy and six unilateral ureteral obstruction (UUO) studies, respectively. The kidney samples of UUO models (26 cases and 22 sham control) were analyzed 7, 10, and 28 days after the surgery [62,63,64,65,66,67]. The diabetic nephropathy models included the db/db type 2 diabetic mice [68,69,70,71] and KKAy [72] or Akita mice [61].

### 2.3. The Most Dysregulated miRNA Signatures in Kidney Diseases

Using RRA and vote-counting analysis, we identified the most dysregulated miRNA signatures in patients with kidney diseases compared to healthy control groups. Each statistical method was used for subgroup analyses.

**Figure 2:**
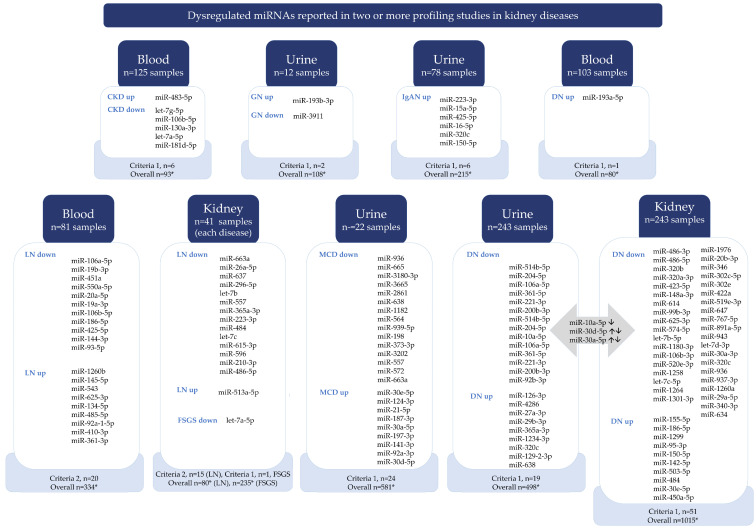
Dysregulated miRNAs reported in two or more profiling studies in kidney diseases. The diagram shows that dysregulated miRNAs have been reported in two or more profiling studies in kidney diseases. The findings were obtained through a vote-counting analysis (subgroup analysis of disease and sample type). Two criteria were used to list miRNAs. The first criterion was applied to all kidney diseases reported in two or more studies, where dysregulated miRNAs had LogFC > 2, FC > 4, and *p* < 0.1. The second criterion was applied to lupus nephritis reported in three or more studies, where dysregulated miRNAs had LogFC > 2, FC > 4, and *p* < 0.1. The detailed results can be found in Tables S1–S11. Abbreviations: *: represents criteria for vote-counting as LogFC > 2, FC > 4, and *p* < 0.1; down: downregulated, up: upregulated; CKD: chronic kidney disease; GN: glomerulonephritis; IgAN: IgA nephropathy; DN: diabetic nephropathy; MCD: minimal change disease; LN: lupus nephritis.

Vote-counting analysis (Figure 2) revealed the most up- and downregulated specific miRNAs in each kidney disease by different sample types. As a result, 71 miRNAs in DN, 24 in MCD, 35 in LN, 1 in FSGS, 6 in IgAN, 6 in CKD, and 2 in GN were the most dysregulated by vote-counting analysis criteria (Figure 2).

For DN, the top upregulated miRNAs were urinary miR-126-3p, miR-193a-5p in blood, and miR-155-5p in kidney tissue, while for LN, they were miR-1260b in blood and miR-513a-5p in kidney tissue. Specific miRNAs were downregulated in DN, including urinary miR-514b-5p and miR-486-3p in kidney tissue. In LN, miR-106a-5p in blood and miR-663a in kidney tissue were downregulated. In FSGS, let-7a-5p in kidney tissue was also downregulated. In MCD, urinary miR-936 was downregulated and miR-30e-5p was upregulated. In IgAN, urinary miR-223-3p was upregulated. The top miRNAs for GN were miR-193b-3p and miR-3911, and for CKD (etiology not defined in the original article) they were miR-483-5p and let-7g-5p.

Certain miRNAs were similarly dysregulated in multiple diseases. For example, miR-320c and miR-150-5p were dysregulated in both DN and IgAN. The miR-10a-5p, miR-30d-5p, and miR-30a-5p were dysregulated in urine and kidney tissue in DN patients (Figure 2). Some miRNAs are dysregulated in different diseases. For instance, miR-936 is downregulated in the kidney tissue of DN and the urine of MCD. Conversely, miR-30e-5p is upregulated in the kidney tissue of DN and the urine of MCD (Figure 2). Additionally, miR-486-5p expression decreased in the kidney tissue of DN and LN patients.

According to the RRA subgroup analysis of disease and sample types, miR-26a-5p was significantly reduced in the kidney tissue of LN, while miR-107 was increased in the blood samples of LN patients. However, no other miRNAs were significantly dysregulated by RRA in both subgroup analyses of disease or disease and sample type. When considering the subgroup analysis of disease types, miR-181a-5p, miR-15a-5p, miR-27a-3p, and miR-27b-3p were significantly dysregulated in patients with CKD (as shown in Supplementary Figure S1). The most dysregulated miRNAs were generally consistent between RRA and vote-counting analyses.

Some miRNA profiling studies could not be pooled for meta-analysis, so they were reviewed systematically. Among them, miR-486-5p, miR-99a-5p, miR-133a-3p, and miR-135b-5p were found to be upregulated and downregulated in various kidney diseases. Other miRNAs showed only one type of dysregulation (either upregulated or downregulated) (refer to Supplementary Table S12).

### 2.4. The Most Dysregulated miRNA Signatures in an Early and Late Stage of CKD

We found only two significantly upregulated miRNAs in the early stage of the CKD group compared to healthy controls by RRA, circulating miR-122-5p and urinary miR-27b-3p (summarized with heat map from vote-counting analysis, Figures S2 and S3). The late CKD stage depicted reduced miR-106b-5p and miR-144-3p in the blood and miR-3180-3p in urine samples (Figures S4 and S5). Kidney tissues in late CKD depicted downregulated miR-486-3p and -5p but overexpressed miR-21-5p and miR-155-5p (Figure S6). There were insufficient data to analyze kidney samples in the early stages of CKD and perform subgroup analysis by disease and sample type.

### 2.5. The Most Dysregulated Renal miRNAs in Murine CKD

**Figure 3:**
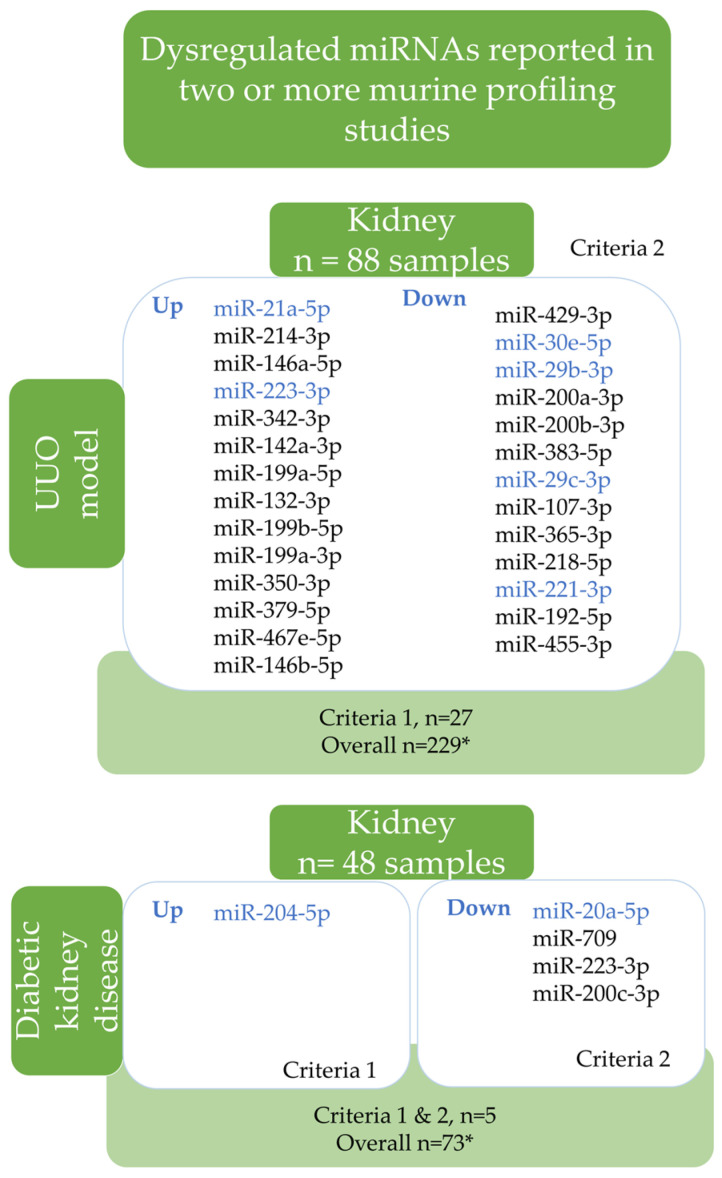
Dysregulated miRNAs reported in two or more murine profiling studies. The diagram shows that dysregulated miRNAs have been reported in two or more profiling studies in murine kidney disease models. The findings were obtained through a vote-counting analysis (subgroup analysis of the disease model). Two criteria were used to list miRNAs. The first criterion was applied to the diabetic kidney disease (DKD) model reported in two or more studies, where dysregulated miRNAs had LogFC > 2, FC > 4, and *p* < 0.1. The second criterion was applied to UUO and DKD models reported in three or more studies, where dysregulated miRNAs had LogFC > 2, FC > 4, and *p* < 0.1. The blue miRNAs indicate an overlap between human CKD and murine CKD models. The detailed results can be found in Tables S13 and S14. Abbreviations: *: represents criteria for vote-counting as LogFC > 2, FC > 4, and *p* < 0.1; down: downregulated, up: upregulated; UUO: unilateral ureteral obstruction; DKD—diabetic kidney disease.

Murine UUO and diabetic nephropathy models were identified among the eligible studies. The most dysregulated miRNA signatures of the murine kidney tissue compared to sham controls identified by vote-counting analysis are summarized in Figure 3. In total, 283 miRNAs were up- and 17 downregulated according to the cut-off values used in vote-counting (*p* < 0.1, Log2FC > 2, FC > 4). Among them, 27 miRNAs in UUO and 5 in the diabetic kidney disease (DKD) model were the most dysregulated (Figure 3). In the UUO model, renal miR-21a-5p, miR-214-3p, and miR-146a-5p were overexpressed, while miR-429-3p, miR30e-5p, and miR-29b-3p were reduced (Figure 3).

Only miR-204-5p was overexpressed, while miR-20a-5p, miR-709, miR-223-3p, and miR-200c-3p were all downregulated in the kidneys of diabetic mice (Figure 3).

### 2.6. Gene Set Enrichment Analysis

Gene set enrichment analyses were conducted for every disease subgroup. The results are presented in a summary data panel, which comprises four parts: the most enriched pathways, the top 20 miRNA-related GO biological processes for each kidney disease, and the top 10 target genes (Figure 4, Figure 5 and Figures S7–S13).

**Figure 4:**
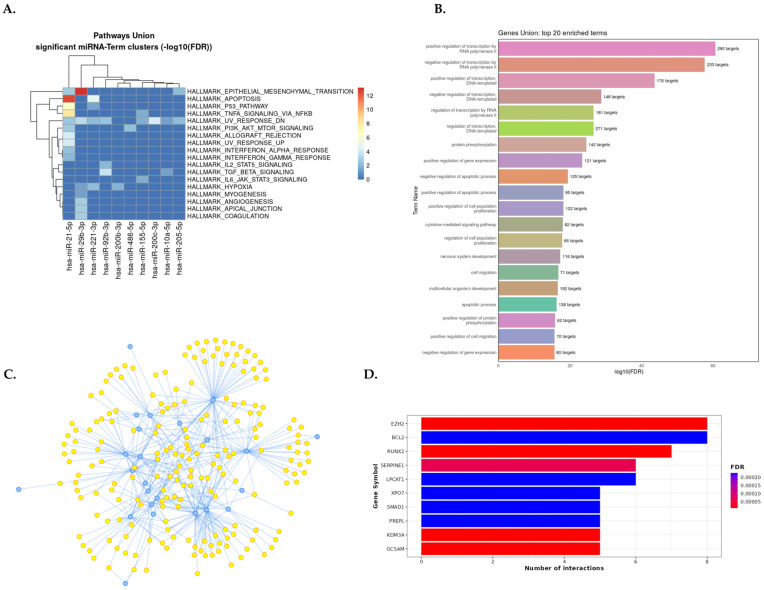
Summary of gene set enrichment analysis of dysregulated miRNAs in diabetic nephropathy (miRNA–DN). (**A**) DIANA–miRPath v4.0 analysis for the pathway union of MSigDB hallmark gene sets of significantly dysregulated miRNA signatures. MSigDB pathway union represents well-defined biological states or processes from the MSigDB 2023.2 release. (**B**) The most strongly enriched 20 GO biological processes related to miRNA–DN from the MIENTURNET web tool. (**C**) Interaction network between miRNAs and target genes from an experimentally validated tool, miRTarBase v8; blue dots represent miRNAs, and yellow dots represent target genes (the raw data are available in Table S15). (**D**) Bar plot with the top 10 target genes on the Y–axis and the number of miRNAs targeting them are shown on the X–axis. The plot is color-coded by increasing the FDR value from red to blue. Abbreviation: DN: diabetic nephropathy.

Enriched MSigDB hallmark gene sets in DN are most significantly associated with epithelial-mesenchymal transition (EMT), apoptosis, hypoxia, myogenesis, angiogenesis, coagulation, several cells’ signaling (p53, IL2/STAT5, TNF-α, TGF-β, IL6, and JAK/STAT3), apical junction, interferon alpha, and gamma response pathways (Figure 4A). The gene set enrichment analysis of dysregulated miRNAs in DN by GO showed the regulation of transcription, the apoptotic process, cytokine-mediated signaling pathway, cell proliferation, and protein phosphorylation as the most significantly enriched biological processes (Figure 4B).

The interaction network between dysregulated miRNAs in DN and their target genes (from an experimentally validated tool, miRTarBase v8) is shown in Figure 4C. As a result, miR-155-5p, miR-200c-3p, miR-21-5p, miR-200b-3p, and miR-29b-3p were the most relevant miRNAs in DN having the highest degree of property (56–32) and eccentricity. The most dysregulated 31 miRNAs had 2113 target genes. Ten top genes targeted by dysregulated miRNAs were EZH2, GCSAM, KDM3A, RUNX2, SERPINE1, BCL2, LPCAT1, PREPL, SMAD1, and XPO7 (Figure 4D). The enriched molecular pathways, GO terms, top target genes, and miR-target gene interaction network in human kidney diseases (CKD, IgAN, LN, MCD, MN, and GN) derived from DIANA-miRPath v4.0 and MIENTURNET are presented as a graph in Figures S7–S13 and results in Table S15–S21.

**Figure 5:**
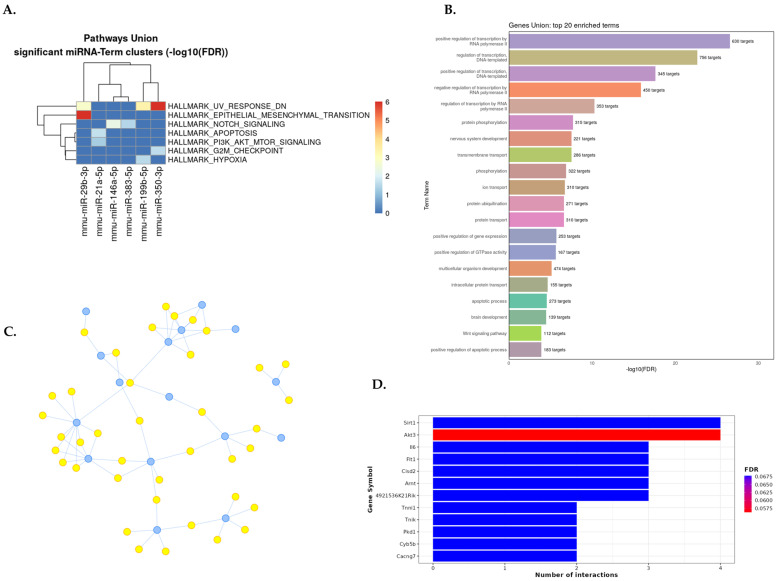
Summary of gene set enrichment analysis of dysregulated miRNAs in murine UUO model (miRNA–UUO). (**A**) DIANA–miRPath v4.0 analysis for the pathway union of MSigDB hallmark gene sets of significantly dysregulated miRNA signatures. MSigDB pathway union represents well-defined biological states or processes from MSigDB 2023.2 release. (**B**) The most strongly enriched 20 GO biological processes related to miRNA–DN from the MIENTURNET web tool. (**C**) Interaction network between miRNAs and target genes from an experimentally validated tool, miRTarBase v8; blue dots represent miRNAs, and yellow dots represent target genes (the raw data are available in Table S22). (**D**) Bar plot with the top 10 target genes on the Y–axis and the number of miRNAs targeting them are shown on the X–axis. The plot is color-coded by increasing the FDR value from red to blue. Abbreviation: UUO: unilateral ureteral obstruction.

In a murine model of renal fibrosis (UUO), EMT, hypoxia, apoptosis, genes downregulated in response to ultraviolet (UV) radiation, G2/M checkpoint of cell division cycle, and cell signaling (Notch and PI3K/Akt/mTOR) pathways were the most enriched in the MSigDB hallmark gene sets analysis (Figure 5A). The gene set enrichment analysis of dysregulated miRNAs in UUO by GO showed the regulation of transcription, the apoptotic process, extracellular matrix organization, GTPase activity, Wnt signaling, cell differentiation, and protein phosphorylation as the most significantly enriched biological processes (Figure 5B).

The interaction network between dysregulated miRNAs in UUO and their target genes is shown in Figure 5C. As a result, miR-29b-3p, miR-29c-3p, miR-200a-3p, miR-21a-5p, and miR-200b-3p were the most relevant miRNAs in UUO, having the highest degree of property (10-6) and eccentricity. Twenty-seven miRNA signatures in UUO are targeted in 164 genes. The ten top genes targeted by dysregulated miRNAs in UUO were Sirt1, Akt3, Il6, Flt1, Cisd2, Arnt, 4921536K21Rik, Tnni1, Tnik, Pkd1, Cyb5b, and Cacng7 (Figure 5D). The gene set enrichment analysis from the murine diabetic kidney disease model is detailed in a graph in Figure S14 and, as a result, in Table S23.

### 2.7. Risk of Bias Assessment

#### 2.7.1. Risk of Bias Assessment in Human CKD Studies

Using MIAME and MIQE tools for array studies, 43.8% of the included studies did not report raw data, and 34.4% did not provide sufficient information about the experimental data processing protocol. For all studies, other key aspects (such as annotation of array design, experiment design and sample annotation, and experiment variables) were fully reported (Supplementary Table S24).

#### 2.7.2. Risk of Bias Assessment in Murine Experimental Models of CKD Studies

All studies had a low or medium risk of bias according to SYRCLE’s RoB tool for animal studies. See Supplementary Table S25 and Figure S15A. Furthermore, all studies were assessed using MIAME and MIQE tools for array studies, which indicated a low risk (see Supplementary Table S24 and Figure S15B).

## 3. Discussion

MiRNAs are dysregulated in kidney diseases and are known to play an essential role in kidney physiology and pathology. Therefore, understanding miRNA dysregulation during CKD is essential to develop new molecular biomarkers and therapeutic agents. Summarizing miRNA profiling studies in CKD can be challenging due to the many factors contributing to heterogeneity. These factors include the patient population, biological sample types, study design, technical platforms, and disease etiology. Combining miRNA profiling studies can also be complicated due to the lack of reported raw data and different platforms with various normalization methods. However, recent statistical approaches have made it possible to combine miRNA profiling studies from different platforms without raw data and generalized normalization using RRA [20,73] and the vote-counting method [21]. These approaches have been successfully validated in previous studies in kidney diseases [13,15,17] and heart failure [22]. Our meta-analysis summarizes hypothesis-free miRNA profiling studies of CKDs by etiology, biological samples, and stages that might reveal novel miRNAs and avoid miRNA selection bias from the investigator side.

According to a vote-counting analysis, 6 miRNAs of CKD, 2 of GN, 6 of IgAN, 71 of DN, 35 of LN, 1 of FSGS, and 24 of MCD were reported in at least two profiling studies and validated. We found only a few significantly dysregulated miRNAs by RRA for a disease and sample type subgroup. We focused solely on hypothesis-free profiling studies that included healthy controls and created subgroups based on each disease and sample type, which might explain the small pool for RRA and the few significantly dysregulated miRNAs. Despite this, both the vote-counting and RRA results were consistent. The enrichment analysis of the top dysregulated miRNAs in each kidney disease revealed their involvement in various pathways associated with CKD pathogenesis, which is discussed in Section 3.2.

### 3.1. The Most Dysregulated miRNA Signatures in Kidney Diseases

We identified eight miRNAs consistent with previous meta-analyses on DN: miR-30d-5p, miR-320c, miR-200c-3p, miR-30a-5p, miR-21-5p, miR-204-5p, miR-27a-3p, and miR-10a-5p [15]. A meta-analysis of LN patients identified five meta-signatures in kidney tissue, nine in blood, and five in urine samples, but only miR-26a-5p, miR-145-5p, and miR-1260b corroborate our results [17].

Fifteen studies have reported miRNA dysregulation in a kidney biopsy. Most studies were conducted on late-stage CKD patients with DN, FSGS, IgAN, LN, and MPGN. Testing miRNAs in kidney tissue could help to estimate the progression rate and pathological changes in glomerular, tubulointerstitial, and/or vascular compartments. However, a kidney biopsy is an invasive and complicated procedure and is unrecommended for all patients. Also, it is limited to identifying atypical histological abnormalities of CKD or sudden kidney functional loss, as eGFR or the urine albumin-to-creatinine ratio does. Therefore, urine miRNAs are preferred for diagnostic purposes rather than miRNAs found in kidney tissue or blood. However, miRNAs might be used as a therapeutic nucleic acid targeted at kidney tissue. Delivery of specific miRNAs to the kidney tissue in murine studies has proven challenging due to adverse effects [74].

Various body fluids contain miRNAs, including blood, urine, feces, saliva, and milk [75]. miRNAs are surprisingly stable in both urine [11] and plasma [12], released from cells either in a passive way (e.g., inflammation and necrosis) or secreted as extracellular vesicles [76] and macromolecular complexes [77,78,79,80] or lipoproteins. Unlike miRNA profiles sequenced from plasma or urinary exosomes, cell-free urine does not contain many miRNAs because of high RNase activity in the kidney, bladder, and urinary tract [76]. Urine miRNAs are either shed from the urinary tract cells [81] or filtered passively from the plasma; thus, these miRNAs could represent either renal or systemic diseases. Nevertheless, the noninvasive sampling method for urinary miRNA assessment has a clear diagnostic advantage, such as determining an unexpected or sudden loss of kidney function by eGFR and creatinine levels.

The upregulated urine miR-27a-3p in CKD patients aligns with studies showing elevated miR-27a-3p in diabetic kidneys [82]. Podocytes overexpress miR-27a in response to high glucose, which represses peroxisome proliferator-activated receptor gamma (PPARγ), leading to the over-activation of the β-catenin signaling and α-SMA, a hallmark of EMT [83]. EMT was one of our results’ most enriched molecular pathways.

The reduced miR-486-5p expression in kidney biopsies of DN patients corroborates murine diabetic nephropathy or ischemic kidney injury studies [84,85]. In mice, miR-486-5p attenuated pulmonary fibrosis by repressing TGF-β signaling [86]. The hyperglycemia-induced miR-486 repression was reported to overexpress its target NFAT5, a transcription factor promoting renal fibrosis through AKT phosphorylation [84]. Similarly, downregulated serum miR-486-5p in DN patients [87] and murine heart failure [88] were reported.

We found upregulated miR-1260b in blood samples of CKD patients [26,49,51], although no previous studies have revealed its direct role in the pathogenesis. Transcription factor YY1 might link miR-1260b to kidney disease as it was reported to regulate cell proliferation and apoptosis in lung cancer via miR-1260b [89]. YY1 was reported to repress TGFB1 in murine DN and human mesangial cells [90], reducing α-SMA and, therefore, EMT in vivo and in vitro [91].

We noticed the miR-30 family among the results, specifically miR-30c, which has been observed in diabetic mice and urine samples of diabetic patients. On the other hand, miR-30e has only been reported previously in mice with kidney fibrosis [92]. However, miR-30e-3p is present in both the urine exosomes and plasma of IgAN patients [44,46]. The upregulation of miR-30e may dampen fibrosis by reducing EMT and TGF-β1 via targeting mitochondrial uncoupling protein 2 (UCP2) in NRK-52E cells [92]. Future cohort studies could test specific miRNAs from our results to confirm their potential for diagnosis, prognosis, and treatment.

Among miRNAs, miR-936 has never been validated in kidney diseases, only reported in miRNA profiling studies in MCD [25,34]. Notably, we found elevated miR-936 expression in human diabetic kidney tissues and high glucose-induced HK-2 human proximal tubular cells (Figure S16). To the best of our knowledge, we have first validated miR-936 in human kidney biopsies and cell cultures. Its role in kidney disease needs further investigations. However, we postulate that miR-936 might participate in EMT as it was identified to suppress cell proliferation and invasion in laryngeal cancer [93].

Several dysregulated miRNAs in our analysis are well known in kidney diseases. For instance, miR-21 was one of the most upregulated in human urine, followed by miR-155-5p in kidney tissue, and both participate in the endothelial-to-mesenchymal transition during human and murine allograft rejection [94].

Besides the mentioned sample types, future research should address the single-cell transcriptomic analysis from single kidney cells. For example, T. Yoshida and colleagues explored podocytes’ urinary single-cell RNA-seq data from FSGS subjects and podocyte cell lines and reported miR-1285-3p as one of the most differentially expressed miRNAs in the APOL1 genotype [95].

Most CKD patients in the early stages remain asymptomatic; increased proteinuria or serum creatinine levels usually appear when significant tissue damage has occurred. Early CKD detection should prevent kidney failure [96] and predict prognosis [97], but most patients are diagnosed late due to the above-mentioned diagnostic limitations. Thus, new and less invasive biomarkers (such as miRNAs in liquid biopsies) are needed to improve early CKD management. Also, miRNAs could serve as modifiable treatment markers as ACE inhibitors or as a β-blocker treatment of hypertensive nephropathy patients with reduced miR-103a-3p and albumin-to-creatinine ratio [19,98].

Therefore, we also investigated stage-specific miRNA signatures of CKD from eligible studies. Only urinary miR-27b-3p and circulating miR-122-5p were upregulated in early-stage CKD patients. The miR-122 upregulation may promote renal tubulointerstitial fibrosis by targeting FOXO3 [99]. There were insufficient data to analyze early-stage kidney biopsies, in line with the concept of avoiding invasive procedures routinely in early kidney disease. In late-stage CKD, circulating miR-106b-5p and miR-144-3p and kidney biopsy-specific miR-486-3p and miR-486-5p were downregulated [93].

Macrophage-derived miR-106b was recently associated with inflammation-induced hypertension in mice [100]. In contrast to our analysis, upregulated miR-144 was found in CKD patients with eGFR < 30 [101]. Interestingly, miR-144 was downregulated in Alb/TGF-β transgenic mice with renal fibrosis [101] and pulmonary fibrosis [102], corroborating our meta-analysis.

Our review revealed repressed miR-451a in the blood and kidney samples at late-stage CKD. Both in vivo and in vitro, miR-451 overexpression inhibits glomerular and mesangial cell proliferation in diabetes [103]. The progressive upregulation of urinary exosome miR-451-5p predicted albuminuria diabetic rats [104]. Urinary miR-451 correlated positively with eGFR but negatively correlated with plasma miR-451 levels and urinary albumin [105]. Thus, miR-451 might be a marker to distinguish early- and late-stage CKD.

Our finding lacks certainty since we have analyzed various types of kidney diseases and sample types in both early and late-stage CKD with small sample sizes. To gain a more comprehensive understanding of miRNA dysregulation during the early stages of kidney diseases, we need to conduct additional cohort studies with larger sample sizes.

We have discovered that miR-204-5p is dysregulated in murine experimental DKD and human DN. However, miR-221-3p, miR-30e-5p, and miR-29b-3p are the miRNAs that overlap with human DN studies and the murine UUO model. On the other hand, miR-200c-3p and miR-709 are only dysregulated in the murine DKD model (refer to Figure 3). miR-21a-5p was found in both human MCD and murine UUO models. These findings are consistent with a previous study on kidney fibrosis [13]. Among them, miR-21 targets Pten, a multifunctional gene that affects cell proliferation, apoptosis, fibrosis, and inflammation [106]. In addition to Pten, miR-21 can target Smad7, a negative regulator of TGF-β1–Smad3 signaling, thus altering several metabolic pathways and contributing to fibrosis. Furthermore, miR-21 has been extensively researched and identified as a viable therapeutic target for acute kidney injury [107] and CKD [108].

### 3.2. miRNA Signature-Related Molecular Pathways

According to the enrichment analysis, miRNA signatures and their target genes can target several pathways that are important in kidney structure and function, such as apoptosis, EMT, hypoxia, inflammatory reactions, G2/M cell cycle arrest, cell signaling (β-catenin, TNF-α, TGF-β, and Notch signaling), and fatty acid metabolism [109,110,111].

After experiencing an injury, the cells in the glomeruli, tubules, and interstitials will undergo excessive synthesis and decreased extracellular matrix degradation. This results in kidney fibrosis involving indigenous cells of the cortex and medulla, as well as inflammatory cells, which is end-stage kidney disease, regardless of the primary cause of the damage. During these processes, several post-transcriptional and transcriptional activations and various signal transduction processes will occur. Several studies on CKDs have reported the miRNA signatures, their target genes, and enriched molecular pathways identified through our meta-analysis. This information may prove helpful in the future for developing targeted therapies and not just for understanding the molecular mechanisms of CKD.

Apoptosis causes a loss of parenchymal cells and affects inflammation, fibrosis, and the immune response [110]. Several miRNAs were associated with apoptosis [106]. For example, miR-486-5p prevents apoptosis in endothelial cells by targeting PTEN [112]. In a mouse model of ischemic kidney injury, miR-486-5p resulted in functional and histologic kidney protection [112]. TNF-α signaling via NF-kB, the second most enriched pathway, and its superfamily cytokines induce apoptosis, attracting death receptor multiprotein complexes [110]. TNF-α binds to the TNF receptor, activating NF-κB [109] and controlling various inflammatory genes that are vital in kidney disease [109]. miRNAs have the potential to interact with canonical and non-canonical pathways of NF-κB. For example, increasing miR-181a levels can reduce CRY1 expression, activating TLR/NF-κB and potentially improving renal fibrosis in rats [113].

We observed that some of the top dysregulated miRNAs were “hypoxia-miRs” which affect the transcription factor hypoxia-inducible factor (HIF) [114]. Our findings in late-stage CKD (Figure S6) and other studies on HK-2 cells [115] support the idea that miR-155 is an essential part of the HIF switch where miR-155 induction leads to an isoform-specific negative feedback loop that ultimately affects HIF-1α activity during prolonged hypoxia [114]. Further research is necessary to comprehend the role of “hypoxia-miRs” concerning kidney diseases.

Fatty acid metabolism-related genes were among the most enriched pathways in CKD, corroborating the clinical observation of dyslipidemia in mild and moderate CKD patients with nephrotic proteinuria [116] and highlighting the role of cholesterol metabolism in kidney disease [111]. Fatty acid or impaired fatty acid oxidation cause mitochondrial overload, leading to kidney damage [117]. Mild-to-moderate CKD patients may have high LDL-C, while moderate-to-advanced CKD patients often have low HDL-C and high triglycerides [117]. Excess cholesterol in cells can cause lipotoxicity, which ultimately worsens renal dysfunction. Adlakha and colleagues reported a dual effect of miRNAs on controlling the genes related to apoptosis and cholesterol homeostasis [118]. Mir-106, miR-27, and miR-122 were the most extensively studied in this regard. Interestingly, we found miR-122 to be the top dysregulated miRNA in the early stages of CKD. This miRNA may be connected to early lipid abnormalities as it controls many genes involved in cholesterol biosynthesis, lipoprotein export, fatty acid oxidation, and synthesis [119,120].

TGF-β1 triggers multiple profibrotic miRNAs in the kidney through Smad signaling, indirectly regulating antifibrotic miRNAs [18]. In our analysis, among the top dysregulated miRNAs, miR-21-5p, miR-27a-3p, miR-155-5p, and miR-106b-5p were enriched in the TGF-β signaling pathway. The miR-21 inhibitor lademirsen has already been tested in a phase II clinical trial, and it ameliorated the kidney function decline in patients with Alport syndrome [121]. However, it is impossible to consider only single miRNAs for complex disease pathology and clinical use. This emphasizes the reason for considering miRNA signatures with the most enriched target genes and pathways in kidney diseases.

### 3.3. Limitations of Our Study

The lack of reported raw data was the major concern for study reproducibility among miRNA profiling studies. However, we applied the biostatistical method to overcome it. Despite complex raw data integration in the analysis, the number of known miRNAs varied with the time the study was performed and the technological platform used, which might lead to heterogeneous results. The number of probes necessary for performing RRA was widely unreported in the original studies. We retrieved it from the manufacturers’ websites for each platform. Furthermore, we compared the miRNA dysregulation in early and late-stage CKD by a meta-analysis of only the disease group because of the limited number of studies, which may lead to inconclusive results related to early- vs. late-stage miRNA dysregulation in CKD.

## 4. Materials and Methods

### 4.1. Search Strategy and Selection Process

Methods were prespecified in a protocol registered with the PROSPERO International Prospective Register of Systematic Reviews (CRD42021283763). No ethical approval was required for this systematic review with meta-analysis, as all data were already published in peer-reviewed journals. No patients were involved in the study’s design, conduct, or interpretation. We performed a meta-analysis of miRNA profiling studies based on the PRISMA 2020 guidelines [122] (Supplementary Table S26) and the *Cochrane Handbook* (version 6.2) [123]. To define our clinical question and eligibility criteria, the PECO framework was applied as follows: the population (P) included patients and murine experimental models for which assays on hypothesis-free whole miRNA profiling were performed; the exposed group (E) included CKD patients regardless of stage or etiology, or murine CKD models irrespective of type; the control group (C) had healthy or sham controls; and the outcome (O) was the identification of dysregulated mature miRNAs in CKD patients by comparison with healthy controls. Searches were conducted up to June 12, 2023, using the strategy specified in Supplementary Methods. There were no language restrictions and filters imposed.

Cochrane Central Register of Controlled Trials (CENTRAL), Web of Science, Embase, Scopus, and MEDLINE (via NCBI PubMed) were screened using the electronic search strategy with following search key: “(microRNAs OR “micro RNA” OR miRNA OR miRs) AND (((diabetes or diabet*) and (kidney or renal)) OR (chronic kidney disease OR “chronic renal” OR “renal insufficiency” OR “kidney fibrosis” OR “renal fibrosis” OR “renal interstitial fibrosis” OR nephropathy OR nephropat* OR nephritis OR nephrit* OR “glomerular sclerosis” OR glomerulosclerosis OR “glomerulus sclerosis” OR “Kimmelstiel-Wilson”))”.

There were no language restrictions and filters imposed. There were 10 human [124,125,126,127,128,129,130,131,132,133] and 12 murine studies [104,134,135,136,137,138,139,140,141,142,143,144] that were ineligible due to the lack of reported data. References were managed in EndNote 20 [Clarivate Analytics, Philadelphia, PA, USA]. After removing automatic and manual duplicates, two independent investigators performed the article selection based on the pre-defined eligibility criteria in a two-step manner—first considering the title and abstract and, subsequently, full-text contents. Cohen’s kappa index was calculated at each selection step. A third investigator solved the disagreements. The reference lists of included studies were also assessed for additional eligible reports.

### 4.2. Eligibility Criteria

The accepted profiling methods were the sequencing of small RNAs, qRT-PCR methods designed for parallel quantification of a large number of miRNAs (96- or 384-well format), or microarray-based methods regardless of array types. Moreover, data on mature miRNA expression must have been provided as fold changes (FCs) or *p*-values.

Studies were excluded from the systematic review if (1) the inclusion criteria were not met; (2) data for outcomes of interest were impossible to extract from the published results and upon request from the corresponding author; (3) studies were conducted on cell lines; (4) the exposed group included acute kidney injury, an idiopathic nephrotic syndrome, CKD with coronary artery calcification, congenital kidney diseases, polycystic kidney disease, and an Alport syndrome; and (5) the control groups included non-healthy individuals like patients with type 2 diabetes or systemic lupus erythematosus (SLE) without CKD. In addition, we excluded studies conducted on genetically modified murine experimental models of kidney disease, on 5/6 nephrectomy, and DOCA-salt-induced hypertension models for murine studies. Regarding study design, observational and interventional studies were eligible, while reviews, case series, and case reports were ineligible.

### 4.3. Data Collection and Synthesis Process

Two independent investigators performed the data collection. Disagreements were solved by consensus. Data were extracted for the following variables: study characteristics, first author, publication year, country, study population, eligibility criteria, the definition of case and control groups, the definition of kidney disease, stages of CKD (as defined in each study either by the histology score or laboratory parameters or eGFR), sample number and type, laboratory parameters, and, when available, serum or urine creatinine, protein, and eGFR. For the results of the miRNA expression, the following variables were extracted: miRNA type, platform, probe number, normalization method, cut-off values, and fold changes or *p*-value. Standardized Microsoft Excel sheets (Microsoft, Office 365, Redmond, WA, USA) were used for data collection.

The mature miRNA names in the original studies were converted into the most recent miRBase release (v.22) [145] by miRBaseConverter [146]. Separate input lists were generated based on the magnitude of miRNA dysregulation by *p*-values, ranking up- and downregulated miRNAs separately. Without *p*-values, miRNAs were ranked by logarithmic fold changes (log2FC) or linear expression values provided in the original article. All technical steps related to the data synthesis process, including the ranking of miRNA lists and creating file formats for the RRA analysis, were performed as previously described [73]. Due to the availability of published data, we considered early-stage kidney disease if the eGFR was more than or equal to 60 mL/min/1.73 m^2^ and late-stage disease if the eGFR was less than 60 mL/min/1.73 m^2^ regardless of disease etiology (eGFR was defined by CKD-EPI [147] or the Japanese GFR equation [148]). For the meta-analysis, the tissue origin recorded in the studies was divided into three groups: the urine group included urinary exosomes or sediment; the blood group included serum, plasma, serum exosomes, and peripheral blood mononuclear lymphocytes (PBMCs); and kidney biopsy was kidney tissue.

In the case of missing data, study authors were contacted for retrieval. If the complete lists of miRNAs were unavailable in the identified eligible reports, we also checked the GEO database. The GEO2R web tool “http://www.ncbi.nlm.nih.gov/geo/info/geo2r.html (accessed on 12 June 2023)” with default options was used to extract the miRNA lists for four human eligible studies [32,35,36,43] and one murine [67] eligible study. The terminology of miRNAs varied with the time the study was conducted. Accession numbers could be used to convert miRNA names to the most current version of miRBase if the miRBase version for each technological platform and study was known.

### 4.4. Meta-Analysis

To identify the most dysregulated miRNA signatures that are consistently up- or downregulated across all studies, we used the Robust Rank Aggregation guideline (RRA) [20,73] and the vote-counting method [21]. The inputs of this method are the normalized ranks, i.e., the achieved ranks divided by the number of probes used in the studies. This method assigns a *p*-value to each element in the aggregated list, indicating how much higher it ranks compared to a null model with random ordering. The advantage of this approach is its robustness to noise, errors, and outliers. In implementing the RRA, we followed the guideline [73], considering only the top miRNAs in the aggregated lists. After Bonferroni *p*-value correction, an adjusted *p*-value lower than 0.05 was considered significant. We also analyzed the key elements of the vote-counting aggregation method to provide additional information [21,149]. For the differentially expressed input lists, we set the following cut-off criteria for both up- and downregulated presences: *p*-value less than 0.1, and in the lack of *p*-value, an absolute fold change larger than 4 (or two-based logarithmic fold change larger than 2). Moreover, we calculated the average of the normalized ranks for each miRNA. We used a heat map to visualize ranking similarities between individual studies and miRNAs [150].

### 4.5. Subgroup Analysis

The expression of miRNAs varies among different species, diseases, and types of tissues. To analyze the dysregulated miRNAs in kidney diseases, we conducted subgroup analyses based on species (human and murine), tissue types (urine, blood, and kidney tissue), and specific diseases (DN, CKD, IgAN, LN, MN, MCD, and FSGS).

Early- and late-stage studies are grouped based on eGFR, considering all kidney diseases to determine stage-specific miRNA dysregulation. Only DN studies are meta-analyzed for the technical platform’s (PCR, microarray, next-generation sequencing) effect on miRNA dysregulation during kidney disease as they have a large enough sample size and are divided into subgroups of blood, urine, and kidney tissue (Figure S17).

### 4.6. Target Gene Prediction and Enrichment Analysis

Overall, RRA and vote-counting results were consistent; thus, we performed gene set enrichment analysis based on the dysregulated miRNAs reported in two or more studies for each kidney disease. The enrichment analysis was performed on GO terms, KEGG pathways, and the Molecular Signatures Database (MSigDB) [151] utilizing DIANA-miRPath v4.0 [152]. miRNA targets were identified by two different algorithms (an experimentally validated tool—miRTarBase (v.8) [153], and an *in silico* target prediction tool—TargetScan (v.8.0)) using default settings. The MIENTURNET web tool is used for network analysis and the top experimentally validated target genes [154].

### 4.7. Risk of Bias Assessment for Individual Studies

The Minimum Information About a Microarray Experiment (MIAME) for array and Minimum Information for Publication of Quantitative Real-time PCR Experiments (MIQE) [155] and Syrcle Rob tools [156] for animal studies were used to assess the study quality.

## 5. Conclusions

MiRNAs are considered novel diagnostic markers and therapeutic agents in kidney diseases. Several miRNA expression profiling studies are conducted using different technological platforms in a variety of kidney diseases in search for new miRNA markers; however, due to the heterogeneity of the study population, methodology, and tissue of origin, it is hard to summarize and identify clinically relevant miRNA markers, specific for the disease and tissue of origin. Our systematic review and meta-analysis found miRNA signatures subgrouped by eight human kidney diseases, two murine models, and three tissues of origin, and their role was confirmed by target genes and enriched molecular pathways, which are already known to be involved in kidney pathogenesis. In our study, the full potential of stage-specific miRNA expression has not been proven, and hence, conducting large prospective cohort studies by validating dysregulated miRNAs in early- and late-stage CKD patients is clinically meaningful. Further experimental studies should also validate the enriched molecular pathways related to miRNA-target genes.

## Figures and Tables

**Table 1:** Characteristics of human miRNA expression profiling studies included in the meta-analysis.

Author, Year	Country	Disease	Sample Type	No. of Samples (Case/ Control)	Assay Type	No. of Probes	Cut-off Criteria for Vote-Counting Analysis ^1^
B. Zapała, 2023 [31]	Poland	DN	urine exosome	8/6	NGS	569	Linear FC < 4, LogFC > 2, *p* > 0.1
C. Beltrami, 2018 [32]	UK	DN	urine	20/20	Microarray	754	
D. Delić, 2016 [24]	Germany	T2DN	urine exosome	8/8	Microarray	2549	
M. Cardenas-Gonzalez, 2017 [33]	USA	DN	urine	58/93	PCR	365	
T. Konta, 2014 [34]	Japan	DN	urine	1/1	Microarray	1257	
Z. Gao, 2020 [35]	China	DN	urine	10/10	Microarray	2549	
F. Conserva, 2019 [36]	Italy	T2DN	kidney tissue	6/4	Microarray	884	Linear FC < 4, LogFC > 2, *p* > 0.1
F. Conserva, 2019 [36]	Italy	T2D-MN	kidney tissue	6/4	Microarray	884	
Y. Pan, 2018 [37]	China	T2DN	kidney tissue	41/20	Microarray	1810	
J. Yu, 2019 [38]	China	DN	kidney tissue	4/4	Microarray	1900	
M. A. Baker, 2017 [39]	USA	DN	kidney tissue	23/14	NGS	428	
F. He, 2014 [40]	China	T2DN	serum	6/6	Microarray	866	Linear FC < 4, LogFC > 2, *p* > 0.1
H. Kim, 2019 [41]	Korea	DN	serum exosome	23/18	NGS	2585	
J. D. Massaro, 2019 [42]	Brazil	T1DN & T2DN	PBMCs	10/40	NGS	1866	
J. Yu, 2019 [38]	China	FSGS	kidney tissue	4/4	Microarray	1900	Linear FC < 4, LogFC > 2, *p* > 0.1
M. A. Baker, 2017 [39]	USA	FSGS	kidney tissue	19/14	NGS	428	
A. Ramezani, 2015 [25]	USA	MCD	urine exosome	5/5	Microarray	173	Linear FC < 4, LogFC > 2, *p* > 0.1
N. Wang, 2015 [43]	China	MCD	urine sediment	4/6	Microarray	2578	
T. Konta, 2014 [34]	Japan	MCD	urine	1/1	Microarray	1257	
Q. H. Min, 2018 [44]	China	IgAN	urine exosome	12/12	NGS	1084	Linear FC < 4, LogFC > 2, *p* > 0.1
N. Wang, 2015 [43]	China	IgAN	urine sediment	18/6	Microarray	2578	
T. Konta, 2014 [34]	Japan	IgAN	urine	1/1	Microarray	1257	
C. C. Szeto, 2019 [45]	China	IgAN	urine	22/6	NanoString	800	
J. Wu, 2018 [46]	China	IgAN	plasma	20/10	PCR	168	Linear FC < 4, LogFC > 2, *p* > 0.1
G. Serino, 2012 [47]	Italy	IgAN	PBMCs	7/7	Microarray	723	
B. Y. Xu, 2020 [48]	China	IgAN	PBMCs	5/4	NGS	1900	
Z. Wang, 2020 [49]	China	IgAN	PBMCs	10/10	NGS	2585	
E. Krasoudaki, 2016 [28]	Greece	LN	kidney tissue	12/3	Microarray	365	Linear FC < 4, LogFC > 2, *p* > 0.1
Y. Dai, 2009 [50]	China	LN	kidney tissue	5/3	Microarray	455	
P. Costa-Reis, 2015 [27]	USA	LN/PSGN	kidney tissue	12/6	NanoString	734	
E. Navarro-Quiroz, 2016 [26]	Columbia	LN	plasma	14/7	NGS	2585	Linear FC < 4, LogFC > 2, *p* > 0.1
A. Flores-Chova, 2023 [29]	Spain	LN	plasma exosome	23/25	NGS	2588	
W. Wang, 2015 [51]	China	LN	blood	8/4	PCR array	372	
M. Ulbing, 2016 [52]	Austria	CKD	serum	10/10	NanoString	800	Linear FC < 4, LogFC > 2, *p* > 0.1
P. Nandakumar, 2017 [23]	USA	CKD	blood	15/15	NGS	347	
X. Liu, 2020 [53]	China	CKD I	serum	15/15	NGS	2585	
X. Liu, 2020 [53]	China	CKD V	serum	30/15	NGS	2585	
T. Konta, 2014 [34]	Japan	CrGN	urine	1/1	Microarray	1257	Linear FC < 4, LogFC > 2, *p* > 0.1
R. Dai, 2023 [50]	China	MsPGN	urine	5/5	NGS	2585	

Footnote: ^1^ represents the cut-off criteria for vote-counting analysis for subgroup analysis of disease and sample type. Abbreviation: NGS: next-generation sequencing; CKD: chronic kidney disease; CKD I: chronic kidney disease, stage I; CKD V: chronic kidney disease, stage V; CrGN: crescentic glomerulonephritis; DN: diabetic nephropathy (type 1 or 2 is not specified in the original article); FC: fold change; FSGS: focal segmental glomerulosclerosis; IgAN: immunoglobulin A nephropathy; LN: lupus nephritis; MCD: minimal change disease; MN: membranous nephropathy; MsPGN: membranoproliferative glomerulonephritis; PSGN: post-streptococcal glomerulonephritis; T1DN: type 1 diabetic nephropathy; T2DN: type 2 diabetic nephropathy; T2D-MN: type 2 diabetes—membranous nephropathy; PBMCs: peripheral blood mononuclear cells.

**Table 2:** Characteristics of murine miRNA expression profiling studies included in the meta-analysis.

Author, Year	Country	Disease Model	Animal	Sample/ Kidney Tissue	No. of Samples (Case/Control)	Assay Type	No of Probes	Cut-off Criteria for Vote-Counting ^1^
Y. Zhang, 2015 [69]	China	db/db mice	Mouse C57BL/KsJ-db/db	tissue	6/6	Microarray	1181	Linear FC < 4, LogFC > 2, *p* > 0.1
J. Long, 2010 [70]	USA	db/db mice	Mouse	tissue	3/3	Microarray	667	
Z. Zhang, 2009 [71]	China	db/db mice	Mouse C57BL/6JLepr-db/db	tissue	9/9	Microarray	568	
H. Ishii, 2021 [61]	Japan	DM	Mouse C57BLKS/J Iar- + Leprdb/ + Leprdb (db/db)	tissue	4/4	Microarray	1881	
H. Ishii, 2021 [61]	Japan	T1DM	Mouse C57BL/6-Ins2Akita/J	tissue	4/4	Microarray	1881	
X. W. Zhu, 2016 [72]	China	T2DM	Mouse KKAy and C57BL/6	tissue	10/10	Microarray	609	
G. Du, 2017 [68]	China	db/db mice	C57BL/Ks	tissue	8/8	NGS	1916	
B. N. Chau, 2012 [62]	USA	UUO-10	Mouse C57BL6	tissue	3/3	Microarray	1003	Linear FC < 4, LogFC > 2, *p* > 0.1
A. C. Chung, 2010 [63]	China	UUO-7	Mouse WT	tissue	8/3	Microarray	375	
F. Glowacki, 2013 [64]	France	UUO-28	Mouse C57BL/6	tissue	4/4	Microarray	567	
R. Bijkerk, 2016 [65]	Netherland	UUO-10	Mouse FoxD1-GC; tdTomato	tissue	3/4	Microarray	384	
R. Morizane, 2014 [66]	Japan	UUO-7	Mouse (ICR)	tissue	4/4	Microarray	627	
K. Yanai, 2020 [67]	Japan	UUO-7	Mouse C57BL/6	tissue	4/4	Microarray	1881	

Footnote. ^1^ represents the cut-off criteria for vote-counting analysis for subgroup analysis of murine CKD model. Abbreviation: NGS: next-generation sequencing; DM: diabetes mellitus; FC: fold change; T1DM: type 1 diabetes mellitus; T2DM: type 2 diabetes mellitus; UUO: unilateral ureteral obstruction, UUO-7: kidney samples collected 7 days after UUO surgery; UUO-10: kidney samples collected 10 days after UUO surgery; UUO-28: kidney samples collected 28 days after UUO surgery.

## Data Availability

All detailed results are available in Supplementary Materials.

## Supplementary Materials

The following supporting information can be downloaded at https://www.mdpi.com/article/10.3390/ncrna10030030/s1, Supplementary File S1; Table S1: Up- and down-regulated miRNAs were reported in kidney tissue of diabetic nephropathy; Table S2: Up- and down-regulated urinary miRNAs were reported in diabetic nephropathy; Table S3: Up- and down-regulated miRNAs were reported in blood samples of diabetic nephropathy; Table S4: Up- and down-regulated miRNAs were reported in blood samples of chronic kidney disease; Table S5: Up- and down-regulated miRNAs were reported in blood samples of IgA nephropathy; Table S6: Up- and down-regulated urinary miRNAs were reported in IgA nephropathy; Table S7: Up- and down-regulated miRNAs were reported in blood samples of lupus nephritis; Table S8: Up- and down-regulated miRNAs were reported in kidney tissue of lupus nephritis; Table S9: Up- and down-regulated urinary miRNAs were reported in glomerulonephritis; Table S10: Up- and down-regulated urinary miRNAs were reported in Minimal change disease; Table S11: Up- and down-regulated miRNAs were reported in kidney tissue of FSGS; Supplementary File S2; Figure S1: Heat map of circulating miRNAs in chronic kidney disease patients as compared to controls; Figure S2: Heat map of circulating miRNAs in early-stage chronic kidney disease patients as compared to controls; Figure S3: Heat map of urinary miRNAs in early-stage chronic kidney disease patients as compared to controls; Figure S4: Heat map of circulating miRNAs late-stage chronic kidney disease patients as compared to controls; Figure S5: Heat map of urinary miRNAs in late-stage chronic kidney disease patients as compared to controls; Figure S6: Heat map of dysregulated miRNAs in renal tissue of late-stage chronic kidney disease patients as compared to controls; Figure S7: Summary of gene set enrichment analysis of dysregulated miRNAs in CKD (disease is not specified in the original studies); Figure S8: Summary of gene set enrichment analysis of dysregulated miRNAs in FSGS; Figure S9: Summary of gene set enrichment analysis of dysregulated miRNAs in glomerulonephritis; Figure S10: Summary of gene set enrichment analysis of dysregulated miRNAs in IgA nephropathy; Figure S11: Summary of gene set enrichment analysis of dysregulated miRNAs in MCD; Figure S12: Summary of gene set enrichment analysis of dysregulated miRNAs in lupus nephritis; Figure S13: Summary of gene set enrichment analysis of dysregulated miRNAs in MN; Figure S14: Summary of gene set enrichment analysis of dysregulated miRNAs in murine model of DKD; Figure S15: (A) Risk of Bias assessment—SYRCLE tool results; (B) Risk of Bias assessment—MIAME and MIQE tools’ results for all species; Figure S16: Validation of miR-936 expression in HK-2 human proximal tubular cell line and in diabetic kidney biopsy samples; Figure S17: Comparison of total dysregulated miRNAs by technical platforms in DN; Table S12: Characteristics of human miRNA expression profiling studies included in the systematic review; Table S24: Risk of Bias assessment—MIAME and MIQE tools results for all species; Table S25: Risk of Bias assessment—SYRCLE’s RoB tool results for murine studies; Table S26: PRISMA 2020 checklist. Supplementary File S3; Table S13: Up- and down-regulated miRNAs were reported in murine model of diabetic kidney disease; Table S14: Up- and down-regulated miRNAs were reported in murine model of diabetic kidney disease; Table S15. Interaction network between miRNAs and target genes in Diabetic nephropathy (DN); Table S16. Interaction network between miRNAs and target genes in Focal segmental glomerulosclerosis (FSGS); Table S17. Interaction network between miRNAs and target genes in Glomerulonephritis (GN); Table S18. Interaction network between miRNAs and target genes in IgA nephropathy (IgAN); Table S19. Interaction network between miRNAs and target genes in Lupus nephritis (LN); Table S20. Interaction network between miRNAs and target genes in Minimal change disease (MCD); Table S21. Interaction network between miRNAs and target genes in Membranous nephropathy (MN); Table S22. Interaction network between miRNAs and target genes in Murine UUO model; Table S23. Interaction network between miRNAs and target genesMurine DKD model.
