# Correction: Garmaa et al. A Systematic Review and Meta-Analysis of microRNA Profiling Studies in Chronic Kidney Diseases. *Non-Coding RNA* 2024, *10*, 30

**DOI:** 10.3390/ncrna11040057

**Published:** 2025-07-30

**Authors:** Gantsetseg Garmaa, Stefania Bunduc, Tamás Kói, Péter Hegyi, Dezső Csupor, Dariimaa Ganbat, Fanni Dembrovszky, Fanni Adél Meznerics, Ailar Nasirzadeh, Cristina Barbagallo, Gábor Kökény

**Affiliations:** 1 Institute of Translational Medicine, Semmelweis University, Nagyvárad tér 4, 1089 Budapest, Hungary; gantsetseg.garmaa@gmail.com (G.G.); ailarr.nz@gmail.com (A.N.); 2 Center for Translational Medicine, Semmelweis University, Üllői út 26, 1085 Budapest, Hungary; stfnbndc@gmail.com (S.B.); samatiok@gmail.com (T.K.); hegyi2009@gmail.com (P.H.); csupor.dezso@gmail.com (D.C.); dembrovszky.f@gmail.com (F.D.); meznerics.fanni@stud.semmelweis.hu (F.A.M.); 3 Department of Pathology, School of Medicine, Mongolian National University of Medical Sciences, Ulan-Bator 14210, Mongolia; dariimaa.ganbat21@gmail.com; 4 Faculty of Medicine, Carol Davila University of Medicine and Pharmacy, Dionisie Lupu Street 37, 020021 Bucharest, Romania; 5 Fundeni Clinical Institute, Fundeni Street 258, 022328 Bucharest, Romania; 6 Division of Pancreatic Diseases, Heart and Vascular Center, Semmelweis University, Baross út 22-24, 1085 Budapest, Hungary; 7 Department of Stochastics, Institute of Mathematics, Budapest University of Technology and Economics, Műegyetem rkp. 3, 1111 Budapest, Hungary; 8 Institute for Translational Medicine, Medical School, University of Pécs, 7624 Pécs, Hungary; 9 Institute of Clinical Pharmacy, University of Szeged, Szikra utca 8, 6725 Szeged, Hungary; 10 Department of Public Health, Graduate School of Medicine, International University of Health and Welfare, Tokyo 107-840, Japan; 11 Department of Dermatology, Venereology and Dermatooncology, Semmelweis University, Mária utca 41, 1085 Budapest, Hungary; 12 Section of Biology and Genetics “G. Sichel”, Department of Biomedical and Biotechnological Sciences, University of Catania, 95123 Catania, Italy; cbarbagallo@unict.it; 13 International Nephrology Research and Training Center, Semmelweis University, Nagyvárad tér 4, 1089 Budapest, Hungary

**Text Correction**

There was an error in the original publication [1]. Reference [100]: “Hu, J.M.; He, L.J.; Wang, P.B.; Yu, Y.; Ye, Y.P.; Liang, L. Antagonist Targeting miR-106b-5p Attenuates Acute Renal Injury by Regulating Renal Function, Apoptosis and Autophagy Via the Upregulation of Tcf4. *Int. J. Mol. Med.* **2021**, *48*, 169.” was retracted on 29 January 2024, prior to the publication of this article. The authors were unaware of the retraction at the time of submission to *ncRNA* and have confirmed that Reference number 100 is not essential to their paper.

A correction has been made to **Discussion**, *The Most Dysregulated miRNA Signatures in Kidney Diseases*, Paragraph 13 and the following sentence was deleted: “The upregulation of miR-106b in rat kidneys subjected to ischemia/reperfusion injury reduced cell proliferation and promoted apoptosis and autophagy by targeting transcription factor 4 (TCF4) [100]”. The order of later references has been adjusted accordingly.

In addition, the corresponding author has a new email address which was updated in the original publication [1].

The authors state that the scientific conclusions are unaffected. This correction was approved by the Academic Editor. The original publication has also been updated.
