# Supplementary material for: A Systematic Review and Meta-Analysis of microRNA Profiling Studies in Chronic Kidney Diseases
Source: Noncoding RNA. 2024 May 3;10(3):30. doi: 10.3390/ncrna10030030 (PMC11130806; doi:10.3390/ncrna10030030)
Supplement: Supplementary file 1 [file ncrna-10-00030-s001.zip › Supplementary File S2.pdf]

SUPPLEMENTARY FILE S2

A systematic review and meta-analysis of microRNA profiling studies in chronic kidney diseases

Gantsetseg Garmaa, Stefania Bunduc, Tamás Kói, Péter Hegyi, Dezső Csupor, Dariimaa Ganbat, Fanni Dembrovszky, Fanni Meznerics, Ailar Nasirzadeh, Cristina Barbagallo, Gábor Kökény

Supplementary Methods, 17 supplementary figures, and 4 supplementary tables.

Abbreviations:

ACE: Angiotensin-converting enzyme; ADPKD: autosomal dominant polycystic kidney disease; AKI: acute kidney injury; AGO: Argonaute protein; CENTRAL: Cochrane Central Register of Controlled Trials; CKD: Chronic kidney disease; CTGF: connective tissue growth factor; CGN: crescentic glomerulonephritis; DN: diabetic nephropathy; DKD: diabetic kidney disease; eGFR: estimated glomerular filtration rate; Egr1: Early growth response factor 1; FAO: fatty acid oxidation; FC: fold changes; FSGS: focal segmental glomerulosclerosis; HIF: Hypoxia-inducible factor; INS: idiopathic nephrotic syndrome; IgAN: IgA nephropathy; IMN: idiopathic membranopathy, KEGG: Kyoto Encyclopedia of Genes and Genomes; KLF6: Kruppel-like factor-6, LN: lupus nephritis; log2FC: logarithmic fold changes, MCD: minimal change disease, miRNA: microRNA; RCC: Renal cell carcinoma; RRA: Robust Rank Aggregation, MIAME: Minimum Information About a Microarray Experiment for array, MIQE: Minimum Information for Publication of Quantitative Real-time PCR Experiments, MGN: membranous glomerulonephropathy; MN: membranous nephropathy; mRNA: messenger RNA, MPGN: membranoproliferative glomerulonephritis, PBMCs: peripheral blood mononuclear lymphocytes; PPARγ: peroxisome proliferator-activated receptor gamma; PSGN: post-streptococcal glomerulonephritis; PRISMA: Preferred Reporting Items for Systematic Reviews and Meta-Analyses; SLE: systemic lupus erythematosus; TCF4: targeting transcription factor 4; TGF-β: Transforming growth factor beta; T1DM: type 1 diabetes mellitus; T1DN: type 1 diabetic nephropathy; T2DN: type 2 diabetic nephropathy; UUO: Unilateral Ureteral Obstruction

Supplementary Methods

Meta-analysis

Risk of bias assessment for individual studies

Two independent investigators assessed the risk of bias in the individual studies. Details are provided in Supplementary Table S24-S25. The Minimum Information About a Microarray Experiment (MIAME) for array and Minimum Information for Publication of Quantitative Real-time PCR Experiments (MIQE) [156] guidelines were used to assess the study quality to assess the following variables: raw data, actual data processing, sample annotation and experiment variables, experiment design, annotation of array design, experimental data processing protocol (Supplementary Table S24). For animal studies, we retrieved Syrcle Rob tools [157] to evaluate variables mentioned in Supplementary Table S25.

Synthesis method

Our meta-analysis followed the published guidelines for RRA [20,73]. As a sensitivity analysis, we also calculated the essential components of the vote-counting method [21] based on the number of appearances and a number of opposite presences. Only mature miRNAs were considered in the present study. For human studies, we created different study pools based on the sample types and each kidney disease; urine (exosomes or sediment), blood (serum, plasma, plasma or serum exosomes and peripheral blood mononuclear lymphocytes (PBMCs)), and kidney tissue. For the murine studies, only two pools with the experimental models of CKD could be generated from eligible studies.

Statistical analysis

We used heat map to visualize the similarities in ranking between individual studies and miRNAs [151]. We used the input matrix's Spearman rank correlation with the average linkage method. In the matrix, a value of 0 indicates that miRNA was not reported or unmet with the criteria used in the vote-counting. A value between 0 and 1 represents up-regulated (determined as 1 minus the normalized rank of the up-regulated miRNA).

A value between 0 and -1 indicates that it was downregulated (determined as normalized rank minus one, i.e., it equals one minus the normalized rank is multiplied by negative one). Hence, the sign of the calculated value indicates whether the miRNA is up- or downregulated, and the closer the absolute value to 1 (or -1), the better the rank of the miRNA.

The R language program (version 4.1.2), “RobustRankAggreg” (v.1.1) and `pheatmap` packages, were used to perform the analysis [20].

Validation of miR-936 in human tubular epithelial cells and human kidney biopsies

Cell culture

HK-2 cells (purchased from the American Type Cell Collection (ATCC, #CRL-2190)) were cultured in T75 flasks in DMEM medium containing 1 g/L glucose (Gibco Thermo, Carlsbad, CA, USA) supplemented with 5% FBS and 10 mg/ml penicillin/streptomycin. Cells were grown at 37°C in a humid atmosphere of 95% air and 5% CO2, then seeded on 6-well plates at 10^5 cells/well density and incubated overnight. Then, glucose (HG, n=3) or mannitol (Mann, n=3) were added (both at 20 mmol/L concentration) to repsective cells and incubated for 24h. Control cells received no treatment (CTL, n=3). Then, cells were harvested with Trizol (Invitrogen, Thermo, USA).

Kidney biopsies

Frozen renal cortex tissues from core biopsy specimens were previously diagnosed in routine pathological examinations for diabetic nephropathy with Kimmelstiel-Wilson nodules (DN, n=3) and were retrieved from the 1st and 2nd Department of Pathology, Semmelweis University (Budapest, Hungary) according to the ethics approval of the Semmelweis University Ethical Board (TUKEB 228/2014). Normal kidney cortex samples (CTL, n=3) were excised from healthy tissue area of nephrectomy samples due to renal cell carcinoma. Approximately 10 mg of frozen kidneys were homogenized in 1 ml Trizol solution (Thermo) and RNA extraction was performed as described below.

RNA extraction and qPCR

Total RNA from Trizol samples was extracted according to the manufacturer's protocol. RNA concentration and purity were assessed on a Nanodrop 2000 (Thermo) and then 1 microgram RNA from each sample was reverse transcribed using the High-capacity cDNA kit (Applied Biosystems, Thermo). Expression of miR-936 and U6 as reference was performed using specific miRCURY primers (Qiagen, MA, USA) and performed on a CFX96 thermal cycler (Bio-Rad Hungary, Budapest, Hungary) in duplicates using the miRCURY LNA SYBR Green PCR kit (Qiagen). Expression of miR-936 was normalized to U6 expression using the $2^{-\Delta\Delta Ct}$ formula and expressed as fold expression relative to a control sample. Expression of miR-936 is presented as mean ± standard deviation (SD).

Statitical analysis

Statistical analysis was performed using SPSS 28.0.0 for Windows (SPSS Inc) and the Independent-Samples Mann-Whitney U test (for kidney biopsies) or Kruskal-Wallis test (for HK-2 cells).

Table S12. Characteristics of human miRNA expression profiling studies included in the systematic review

Author, year	Country	Disease	Sample type	No. of samples (case/control)	Assay type	No. of probes	Top 3 upregulated miRNAs	Top 3 downregulated miRNAs
A. Ramezani, 2015	USA	FSGS	urine exosome	16/5	Microarray	1733	miR-373, miR-4801, miR-3145-5p	miR-4466, miR-4728-5p, miR-3175
W. Zhang, 2014	China	FSGS	urine	9/11	Microarray	754	miR-135b-5p, miR-490-3p, miR-208b-3p	NA
A. Ramezani, 2015	USA	FSGS	plasma exosome	16/5	Microarray	1733	miR-455-3p, miR-3065-5p, miR-3065-5p	miR-936, miR-4728-5p, miR-4484
I. O. Sun, 2022	Korea	FSGS/MCD	serum exosome	21/20	NGS	2585	NA	miR-340-3p, miR-1229-3p, miR-99b-5p
A. Ramezani, 2015	USA	MCD	plasma exosome	5/5	Microarray	1733	miR-4327, miR-371a-5p, miR-4667-3p	miR-4758-5p, miR-4530, miR-4640-5p
J. Yu, 2019	China	MCD	kidney tissue	4/4	Microarray	1900	miR-205-3p, miR-4531, miR-3189-5p	miR-130b-3p, miR-204-3p, miR-943
M. A. Baker, 2017	USA	IgAN	kidney tissue	18/14	NGS	428	miR-1273g-3p, miR-1303, miR-99a-5p	miR-451a, miR-486-3p, miR-486-5p (proximal tubules), miR-3182, miR-486-5p (glomeruli)
A. Tripathy, 2023	India	IgAN	kidney tissue	6/6	NGS	2588	miR-21-5p, miR-146b-5p, miR-155-5p	miR-139-3p, miR-127-3p, miR-99a-5p
M. Cardenas-Gonzalez, 2017	USA	LN	urine	89/119	PCR	365	miR-671-5p, miR-30a-5p, miR-2467-3p	miR-3201
R. Khurana, 2017	Austria	CKD	urine exosome	15/10	NGS	360	miR-27a-3p, miR-27b-3p, miR-744-5p (CKD I), miR-126-5p, miR-25-3p, miR-27a-3p (CKD II), miR-744-5p, miR-5100, miR-877-3p (CKD III), miR-27a-3p, miR-27b-3p, miR-5100 (CKD V)	miR-181a-5p, miR-183-5p, miR-122b-3p (CKD I), miR-181a-5p, miR-133a-3p, miR-133b (CKD II), miR-181a-5p, miR-133a-3p, miR-15a-5p (CKD III), miR-181a-5p, miR-181c-5p, miR-96-5p (CKD V)
N. Wang, 2015	China	MN	urine sediment	4/6	Microarray	2578	miR-214-3p, miR-337-5p, miR-2276-3p	miR-665, miR-4793-3p, miR-4440
J. Zhang, 2020	China	IMN	urine	6/5	NGS	836	miR-1180-3p, miR-151b, miR-191-5p	miR-197-3p, miR-132-3p, miR-92b-3p
W. Chen, 2014	China	MN	blood	30/30	Microarray	455	miR-486-5p, miR-133a-3p, miR-204-5p	miR-217-5p, miR-200c-3p, miR-216a-5p
I. O. Sun, 2022	Korea	IMN	serum exosome	19/20	NGS	2585	NA	miR-340-3p, miR-1229-3p, miR-99b-5p
M. A. Baker, 2017	USA	MPGN	kidney tissue	19/14	NGS	428	miR-155-5p, miR-509-3p, miR-4454 (proximal tubule), miR-1290, miR-146b-5p, miR-589-5p (glomeruli)	miR-486-3p, miR-451a, miR-184 (proximal tubules), miR-486-3p, miR-486-5p, miR-451a (glomeruli),
C. Barbagallo, 2019	Italy	MGN	kidney tissue	4/4	Microarray	754	miR-423-5p, let-7b-5p, miR-15b-5p	miR-129-2-3p, miR-135b-5p, miR-33a-5p

Footnote: NA: not applicable, there are no up- or down-regulated miRNAs. Abbreviation: NA: not applicable; NGS: Next-generation sequencing; CKD: chronic kidney disease; CKD I: chronic kidney disease, stage I; CKD V: chronic kidney disease, stage V; FSGS: Focal segmental glomerulosclerosis; G –glomeruli; IgAN: Immunoglobulin A nephropathy; IMN: Idiopathic membranous nephropathy; LN: Lupus nephritis; MCD: Minimal change disease; MN: Membranous nephropathy; MPGN: Membranoproliferative glomerulonephritis; PSGN: Post streptococcus glomerulonephritis; PT – proximal tubule; T1DN: type 2 diabetic nephropathy; T2DN: type 2 diabetic nephropathy; T2D-MN: type 2 diabetes - membranous nephropathy; PBMCs: peripheral blood mononuclear lymphocytes

Supplementary Figure S1. Heat map of circulating miRNAs in chronic kidney disease patients as compared to controls

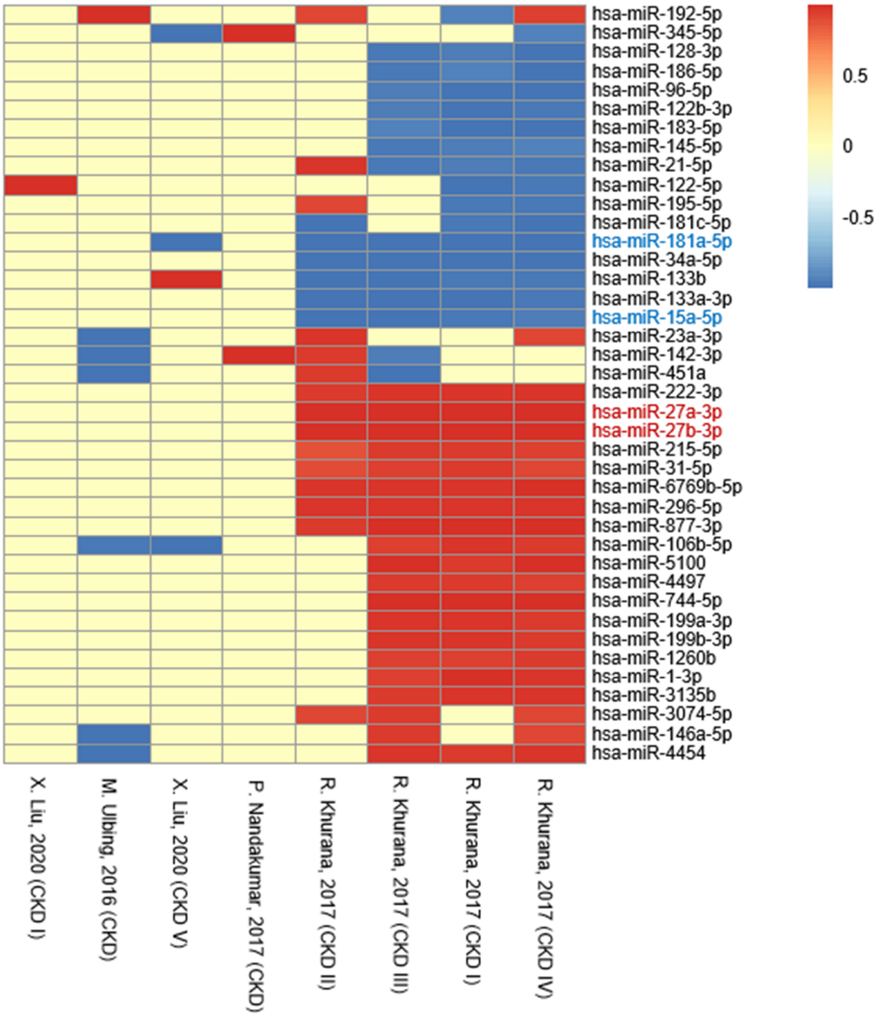

Footnote: The heat map illustrates the dysregulated miRNAs of CKD patients compared to controls according to the heat map of the eight identified eligible studies using an average ranking score. The listed miRNAs were reported in at least three expression profiling studies. Every row corresponds to an individual miRNA, and each column indicates an individual study. The three different colors represent the presence and dysregulation direction of individual miRNAs ranging from 1 to -1 as follows: absent (0 -yellow), up-regulated (0.1 to 1 -red), and downregulated (-0.1 to -1 -blue). The names of the significantly dysregulated miRNAs in Robust Rank Aggregation are colored in blue (downregulated) and red (up-regulated), respectively. If the opposite direction of the individual miR exists, it is provided as blue or red in the corresponding row. Abbreviation: miRNA - microRNA

Supplementary Figure S2. Heat map of circulating miRNAs in an early stage of chronic kidney disease patients as compared to controls

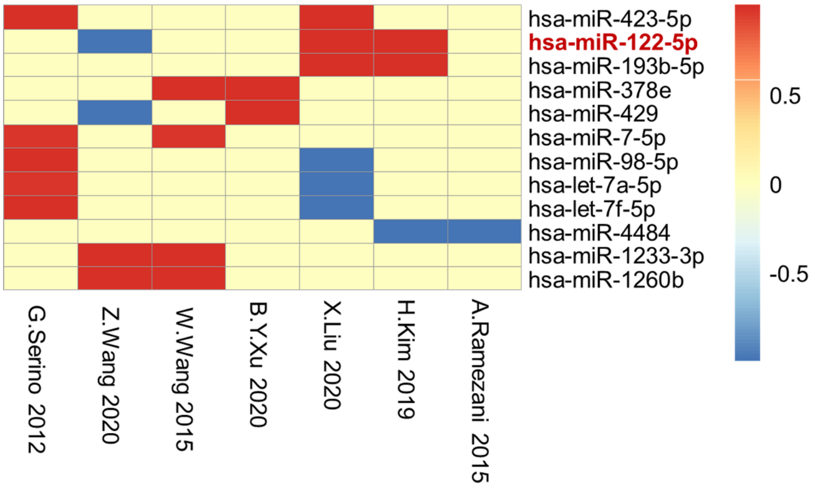

Footnote: The heat map illustrates the dysregulated circulating miRNAs of early-stage of CKD patients compared to controls according to the heat map of the seven identified eligible studies using an average ranking score. The listed miRNAs were reported in at least two expression profiling studies. Every row corresponds to an individual miRNA, and each column indicates an individual study. The three different colors represent the presence and dysregulation direction of individual miRNAs ranging from 1 to -1 as follows: absent (0 -yellow), up-regulated (0.1 to 1 -red), and downregulated (-0.1 to -1 -blue). The names of the significantly dysregulated miRNAs in Robust Rank Aggregation are colored in blue (downregulated) and red (up-regulated), respectively. If the opposite direction of the individual miR exists, it is provided as blue or red in the corresponding row. Abbreviation: miRNA - microRNA

Supplementary Figure S3. Heat map of urinary miRNAs in an early stage of chronic kidney disease patients as compared to controls

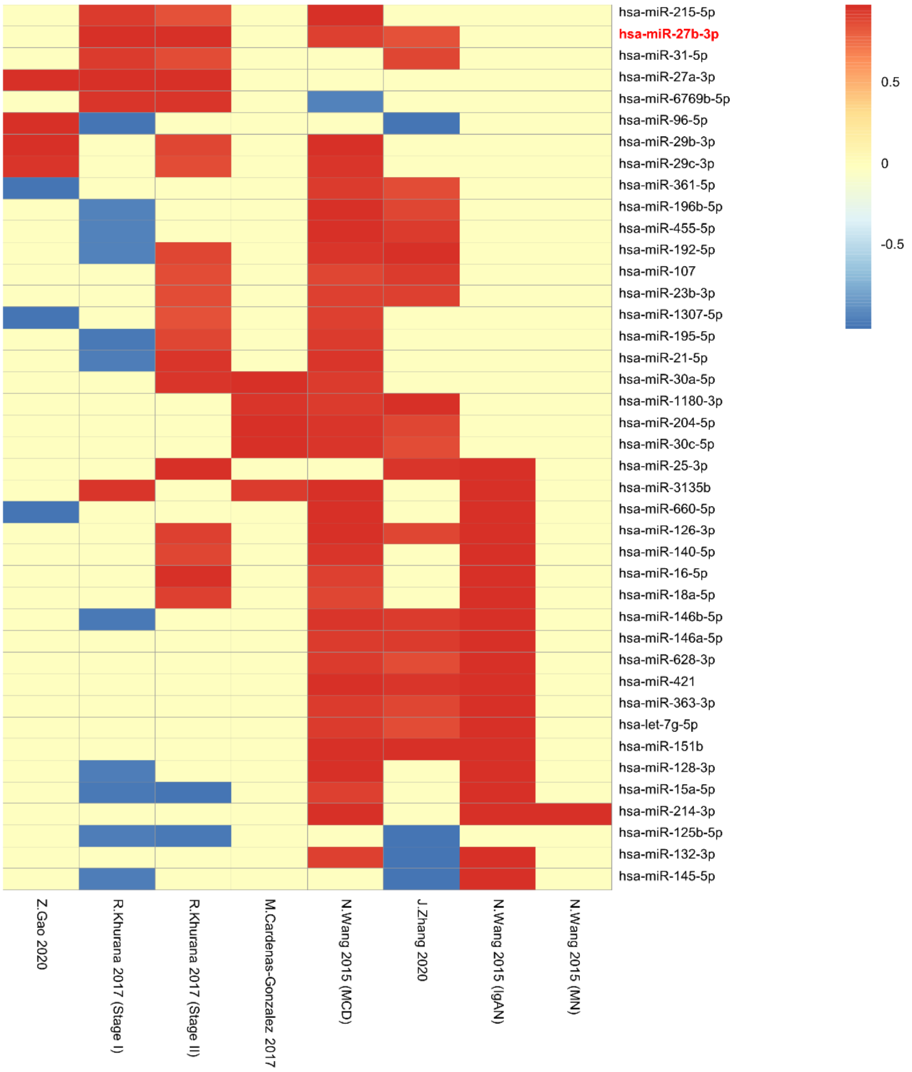

Footnote: The heat map illustrates the dysregulated urinary miRNAs of early-stage of CKD patients compared to controls according to the heat map of the five identified eligible studies (8 individual results) using an average ranking score. The listed miRNAs were reported in at least three expression profiling studies. Every row corresponds to an individual miRNA, and each column indicates an individual study. The three different colors represent the presence and dysregulation direction of individual miRNAs ranging from 1 to -1 as follows: absent (0 -yellow), up-regulated (0.1 to 1 -red), and downregulated (-0.1 to -1 -blue). The names of the significantly dysregulated miRNAs in Robust Rank Aggregation are colored in blue (downregulated) and red (up-regulated), respectively. If the opposite direction of the individual miR exists, it is provided as blue or red in the corresponding row. Abbreviation: miRNA - microRNA

Supplementary Figure S4. Heat map of circulating miRNAs in late stage of chronic kidney disease patients as compared to controls

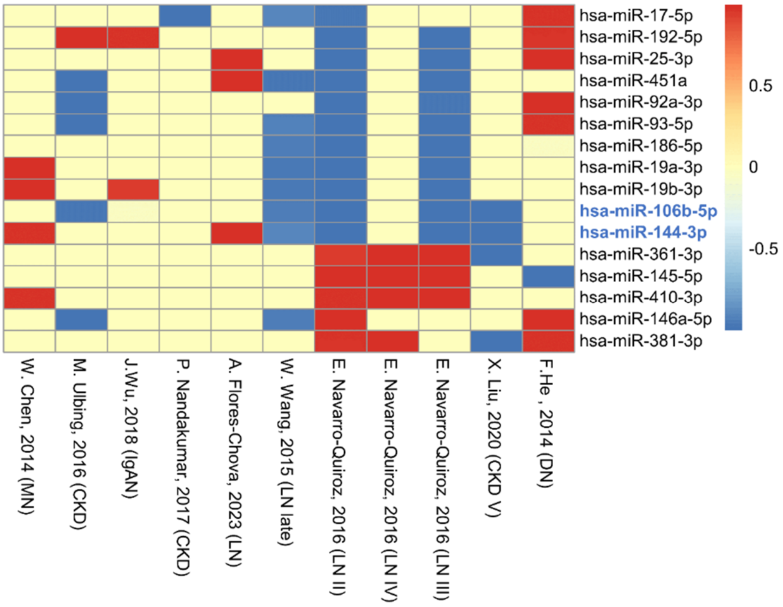

Footnote: The heat map illustrates the dysregulated circulating miRNAs of late-stage of CKD patients compared to controls according to the heat map of the nine identified eligible studies (11 individual results) using an average ranking score. The listed miRNAs were reported in at least four expression profiling studies. Every row corresponds to an individual miRNA, and each column indicates an individual study. The three different colors represent the presence and dysregulation direction of individual miRNAs ranging from 1 to -1 as follows: absent (0 -yellow), up-regulated (0.1 to 1 -red), and downregulated (-0.1 to -1 -blue). The names of the significantly dysregulated miRNAs in Robust Rank Aggregation are colored in blue (downregulated) and red (up-regulated), respectively. If the opposite direction of the individual miR exists, it is provided as blue or red in the corresponding row. Abbreviation: miRNA - microRNA

Supplementary Figure S5. Heat map of urinary miRNAs in late-stage chronic kidney disease patients as compared to controls

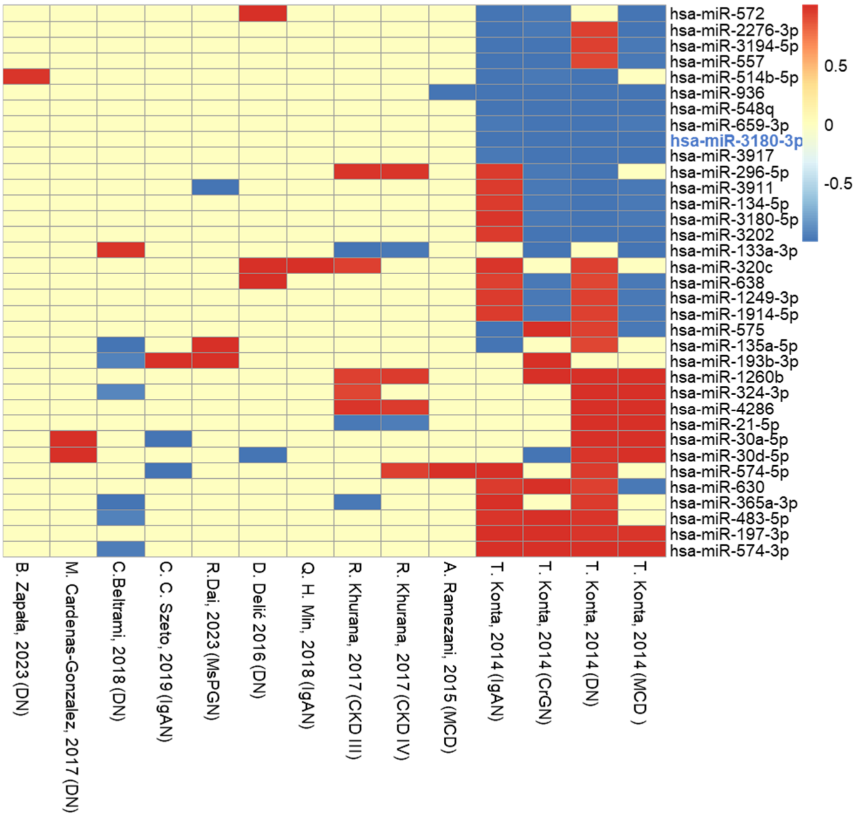

Footnote: The heat map illustrates the dysregulated urinary miRNAs of late-stage CKD patients compared to controls according to the heat map of the ten identified eligible studies (14 individual results) using an average ranking score. The listed miRNAs were reported in at least four expression profiling studies. Every row corresponds to an individual miRNA, and each column indicates an individual study. The three different colors represent the presence and dysregulation direction of individual miRNAs ranging from 1 to -1 as follows: absent (0 -yellow), up-regulated (0.1 to 1 -red), and downregulated (-0.1 to -1 -blue). The names of the significantly dysregulated miRNAs in Robust Rank Aggregation are colored in blue (downregulated) and red (up-regulated), respectively. If the opposite direction of the individual miR exists, it is provided as blue or red in the corresponding row. Abbreviation: miRNA - microRNA

Supplementary Figure S6. Heat map of dysregulated miRNAs in renal tissue of late-stage of chronic kidney disease patients as compared to controls

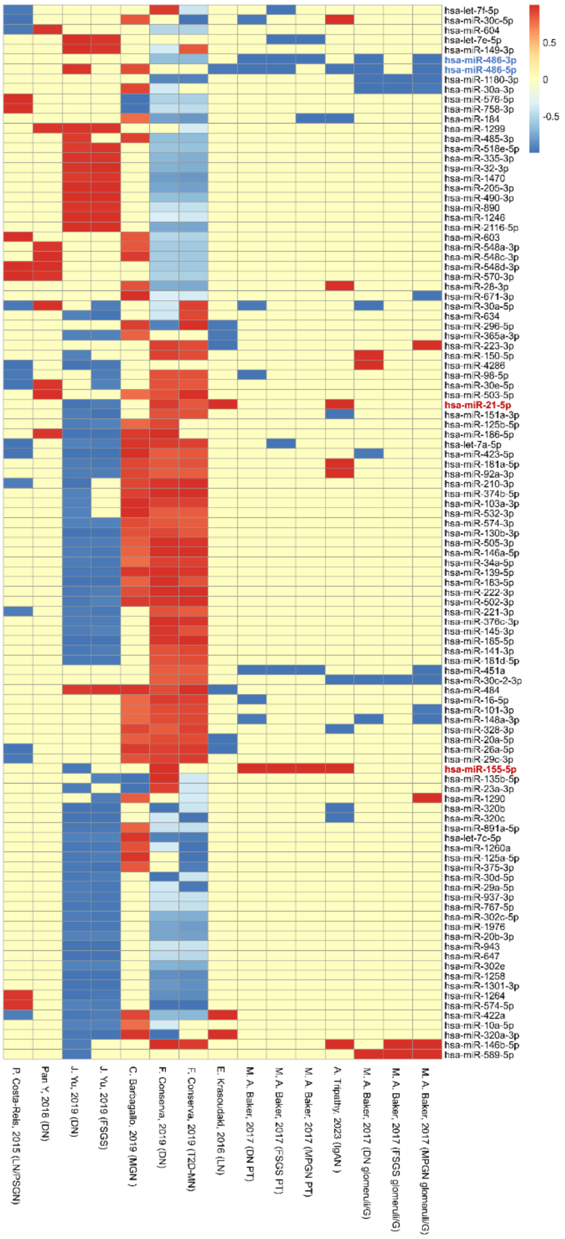

Footnote: The heat map illustrates the dysregulated renal tissue-specific miRNAs of late-stage CKD patients compared to controls according to the heat map of the 8 identified eligible studies (15 individual results) using an average ranking score. The listed miRNAs were reported in at least four expression profiling studies. Every row corresponds to an individual miRNA, and each column indicates an individual study. The three different colors represent the presence and dysregulation direction of individual miRNAs ranging from 1 to -1 as follows: absent (0 -yellow), up-regulated (0.1 to 1 -red), and downregulated (-0.1 to -1 -blue). The names of the significantly dysregulated miRNAs in Robust Rank Aggregation are colored in blue (downregulated) and red (up-regulated), respectively. If the opposite direction of the individual miR exists, it is provided as blue or red in the corresponding row. Abbreviation: miRNA - microRNA

Supplementary Figure S7. Summary of gene set enrichment analysis of dysregulated miRNAs in CKD (disease is not specified in the original studies)

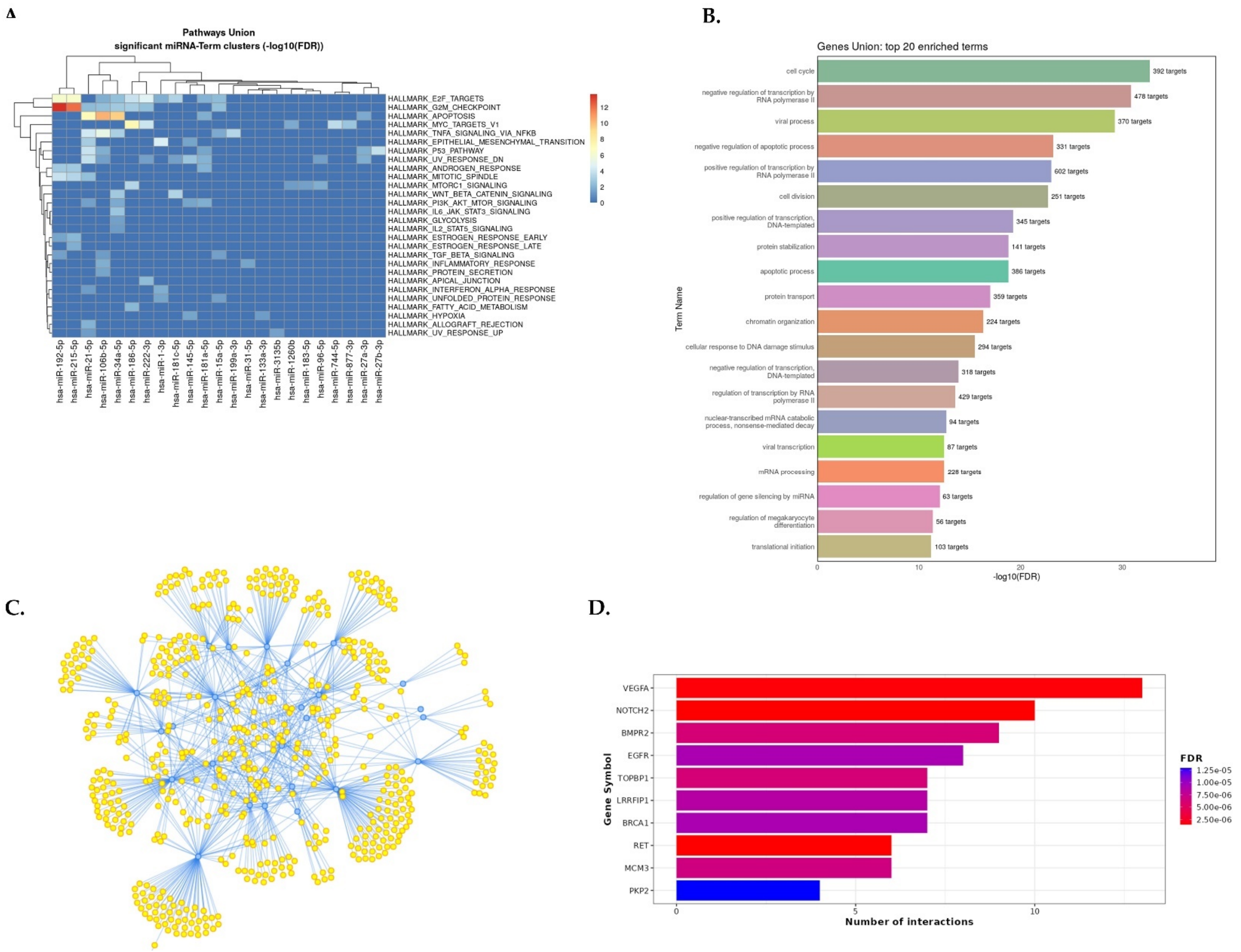

Footnote. Summary of gene set enrichment analysis of dysregulated miRNAs in CKD (miRNA-CKD). A. DIANA-miRPath v4.0 analysis for the pathway union of MSigDB hallmark gene sets of significantly dysregulated miRNA signatures. MSigDB pathway union represents well-defined biological states or processes from MSigDB 2023.2 release. **B.** The most strongly enriched 20 GO biological proceses related to miRNA-DN from the MIENTURNET web tool. **C.** Interaction network between miRNAs and target genes; blue dots represent miRNAs, and yellow dots represent target genes. **D**. Bar plot with the top 10 target genes on the Y-axis and the number of miRNAs targeting them are shown on the X-axis. The plot is color-coded by increasing the FDR value from red to blue. Abbreviation: CKD: chronic kidney disease.

Supplementary Figure S8. Summary of gene set enrichment analysis of dysregulated miRNAs in FSGS

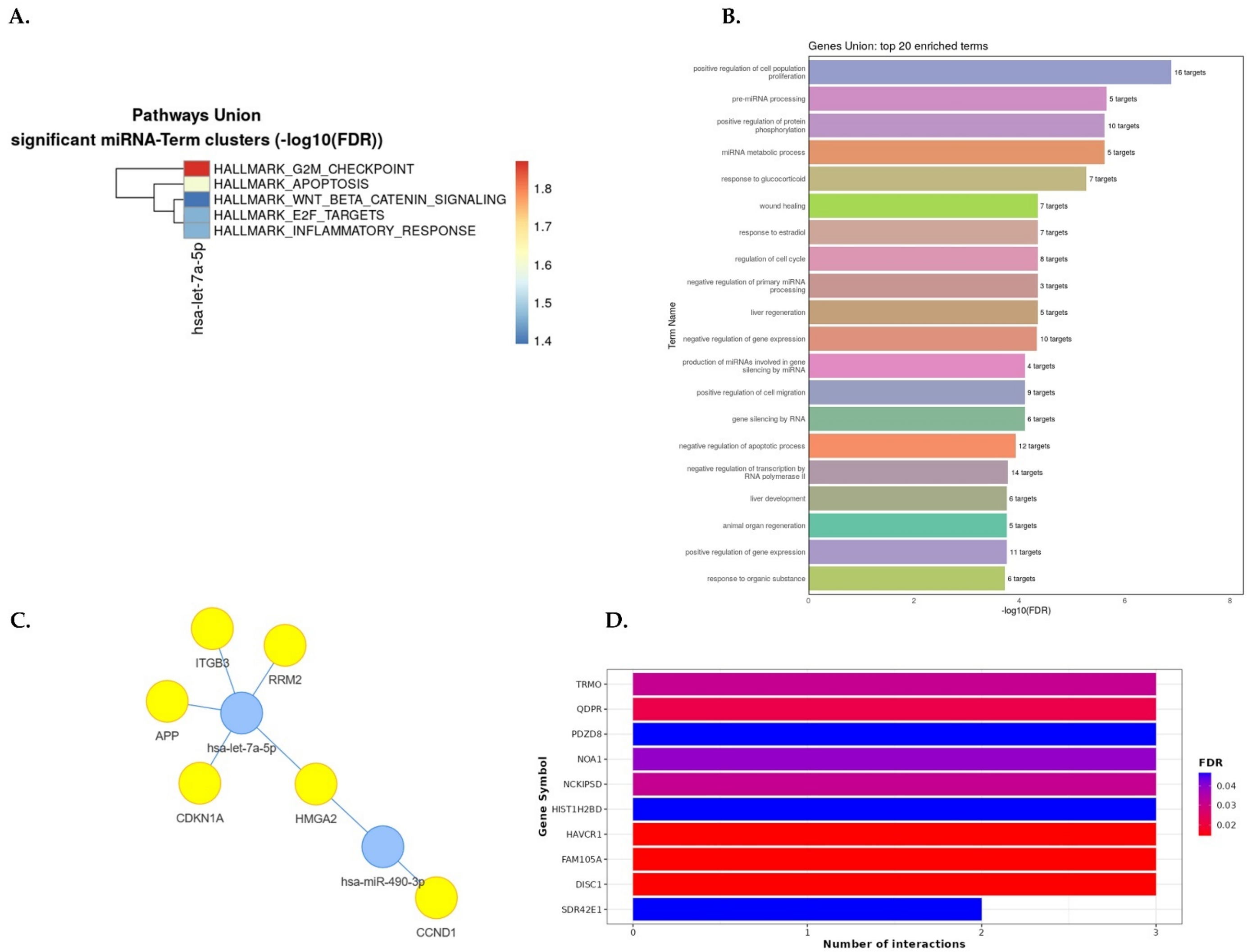

Footnote. Summary of gene set enrichment analysis of dysregulated miRNAs in FSGS (miRNA-FSGS). **A**. DIANA-miRPath v4.0 analysis for the pathway union of MSigDB hallmark gene sets of significantly dysregulated miRNA signatures. MSigDB pathway union represents well-defined biological states or processes from MSigDB 2023.2 release. **B.** The most strongly enriched 20 GO biological process related to miRNA-DN from the MIENTURNET web tool. **C.** Interaction network between miRNAs and target genes; blue dots represent miRNAs, and yellow dots represent target genes. **D**. Bar plot with the top 10 target genes on the Y-axis and the number of miRNAs targeting them are shown on the X-axis. The plot is color-coded by increasing the FDR value from red to blue. Abbreviation: FSGS: Focal segmental glomerulosclerosis.

Supplementary Figure S9. Summary of gene set enrichment analysis of dysregulated miRNAs in Glomerulonephritis

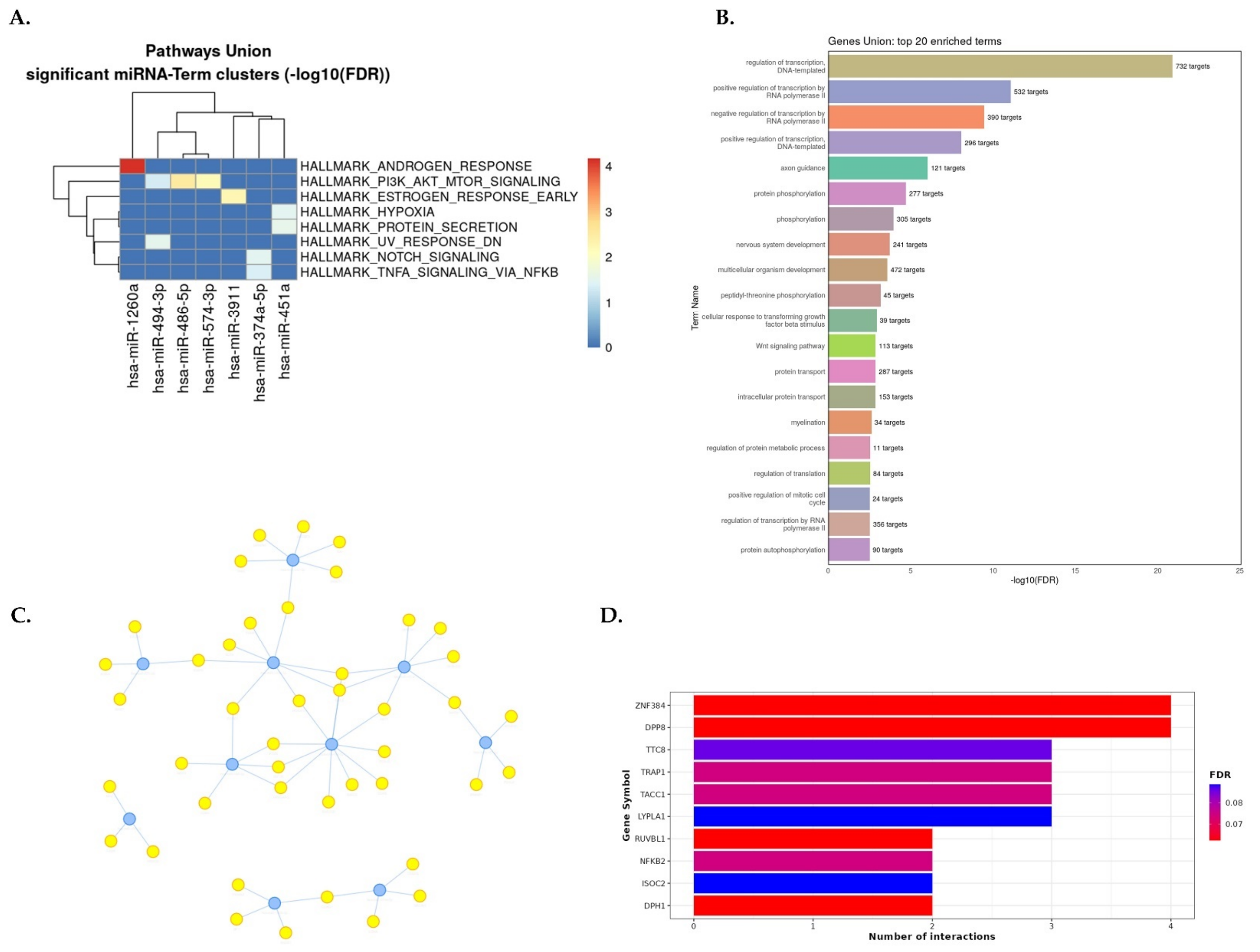

Footnote. Summary of gene set enrichment analysis of dysregulated miRNAs in GN (miRNA-GN). A. DIANA-miRPath v4.0 analysis for the pathway union of MSigDB hallmark gene sets of significantly dysregulated miRNA signatures. MSigDB pathway union represents well-defined biological states or processes from MSigDB 2023.2 release. **B.** The most strongly enriched 20 GO biological process related to miRNA-DN from the MIENTURNET web tool. **C.** Interaction network between miRNAs and target genes; blue dots represent miRNAs, and yellow dots represent target genes. **D**. Bar plot with the top 10 target genes on the Y-axis and the number of miRNAs targeting them are shown on the X-axis. The plot is color-coded by increasing the FDR value from red to blue. Abbreviation: GN: glomerulonephritis.

Supplementary Figure S10. Summary of gene set enrichment analysis of dysregulated miRNAs in IgA nephropathy

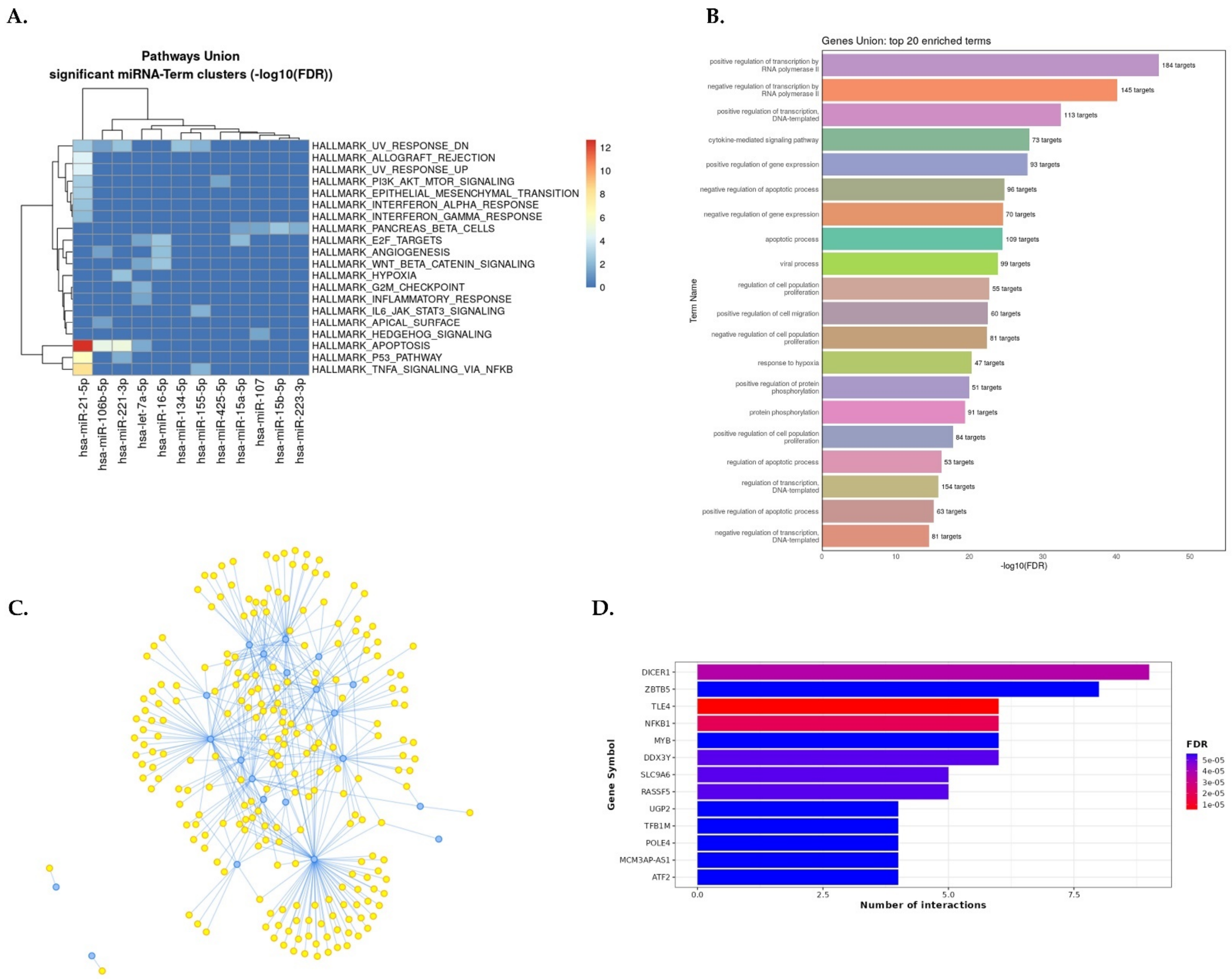

Footnote. Summary of gene set enrichment analysis of dysregulated miRNAs in IgAN (miRNA-IgAN). A. DIANA-miRPath v4.0 analysis for the pathway union of MSigDB hallmark gene sets of significantly dysregulated miRNA signatures. MSigDB pathway union represents well-defined biological states or processes from MSigDB 2023.2 release. **B.** The most strongly enriched 20 GO biological process related to miRNA-DN from the MIENTURNET web tool. **C.** Interaction network between miRNAs and target genes; blue dots represent miRNAs, and yellow dots represent target genes. **D**. Bar plot with the top 10 target genes on the Y-axis and the number of miRNAs targeting them are shown on the X-axis. The plot is color-coded by increasing the FDR value from red to blue. Abbreviation: IgAN: IgA nephropathy.

Supplementary Figure S11. Summary of gene set enrichment analysis of dysregulated miRNAs in MCD

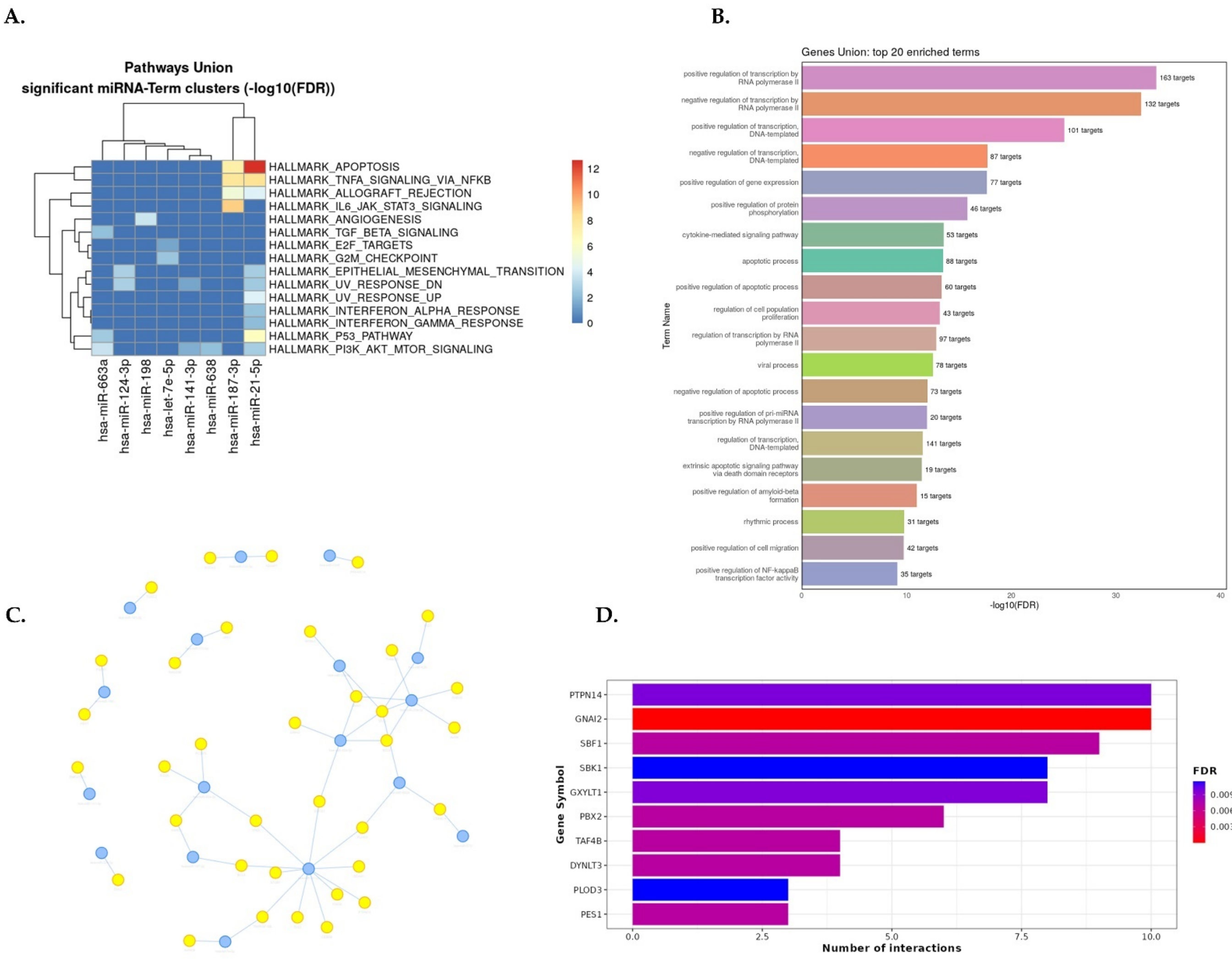

Footnote. Summary of gene set enrichment analysis of dysregulated miRNAs in MCD (miRNA-MCD). A. DIANA-miRPath v4.0 analysis for the pathway union of MSigDB hallmark gene sets of significantly dysregulated miRNA signatures. MSigDB pathway union represents well-defined biological states or processes from MSigDB 2023.2 release. **B.** The most strongly enriched 20 GO biological process related to miRNA-DN from the MIENTURNET web tool. **C.** Interaction network between miRNAs and target genes; blue dots represent miRNAs, and yellow dots represent target genes. **D**. Bar plot with the top 10 target genes on the Y-axis and the number of miRNAs targeting them are shown on the X-axis. The plot is color-coded by increasing the FDR value from red to blue. Abbreviation: MCD: Minimal change disease.

Supplementary Figure S12. Summary of gene set enrichment analysis of dysregulated miRNAs in Lupus nephritis

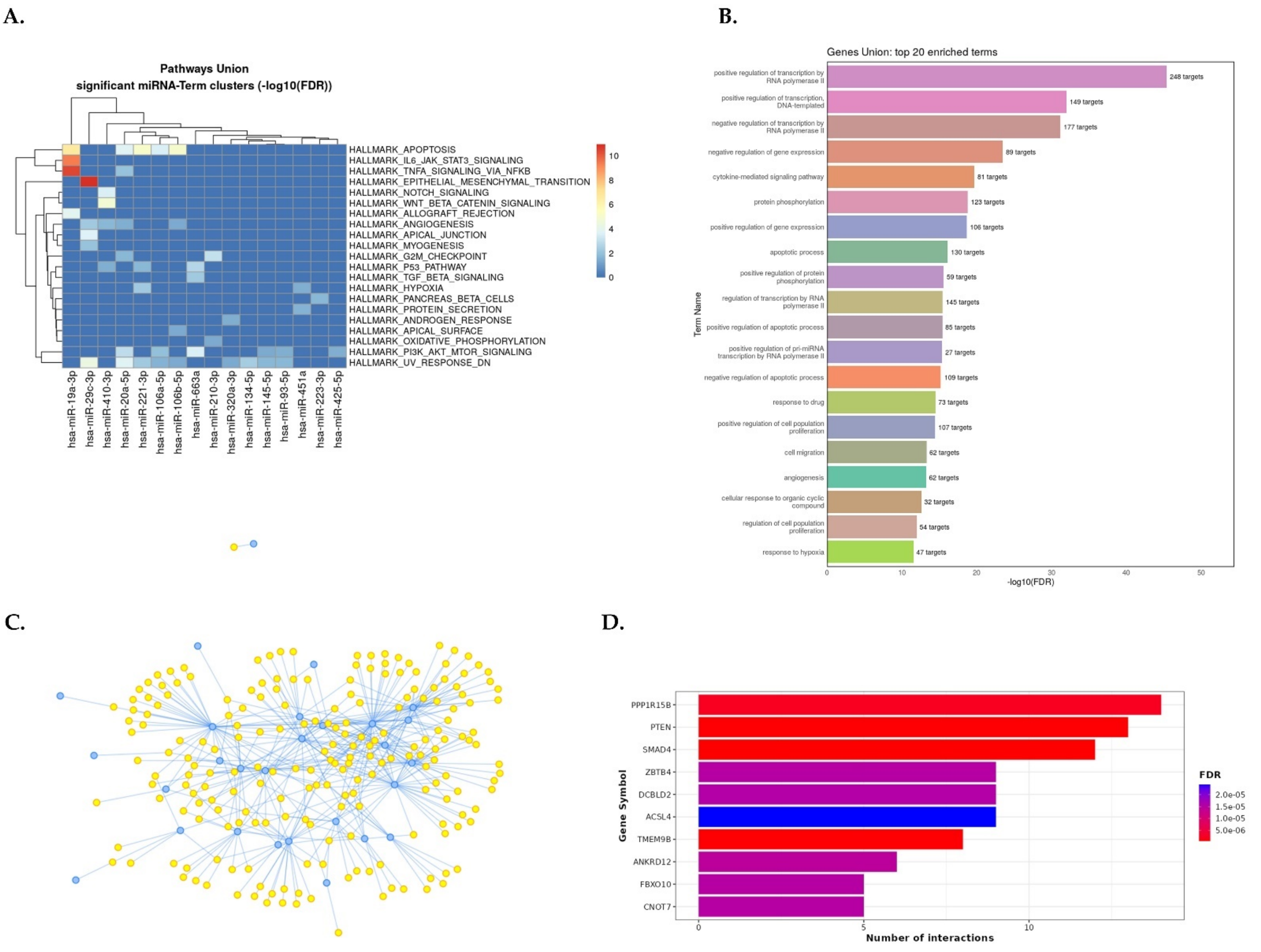

Footnote. Summary of gene set enrichment analysis of dysregulated miRNAs in LN (miRNA-LN). A. DIANA-miRPath v4.0 analysis for the pathway union of MSigDB hallmark gene sets of significantly dysregulated miRNA signatures. MSigDB pathway union represents well-defined biological states or processes from MSigDB 2023.2 release. **B.** The most strongly enriched 20 GO biological proceses related to miRNA-DN from the MIENTURNET web tool. **C.** Interaction network between miRNAs and target genes; blue dots represent miRNAs, and yellow dots represent target genes. **D**. Bar plot with the top 10 target genes on the Y-axis and the number of miRNAs targeting them are shown on the X-axis. The plot is color-coded by increasing the FDR value from red to blue. Abbreviation: LN: Lupus nephritis.

Supplementary Figure S13. Summary of gene set enrichment analysis of dysregulated miRNAs in MN

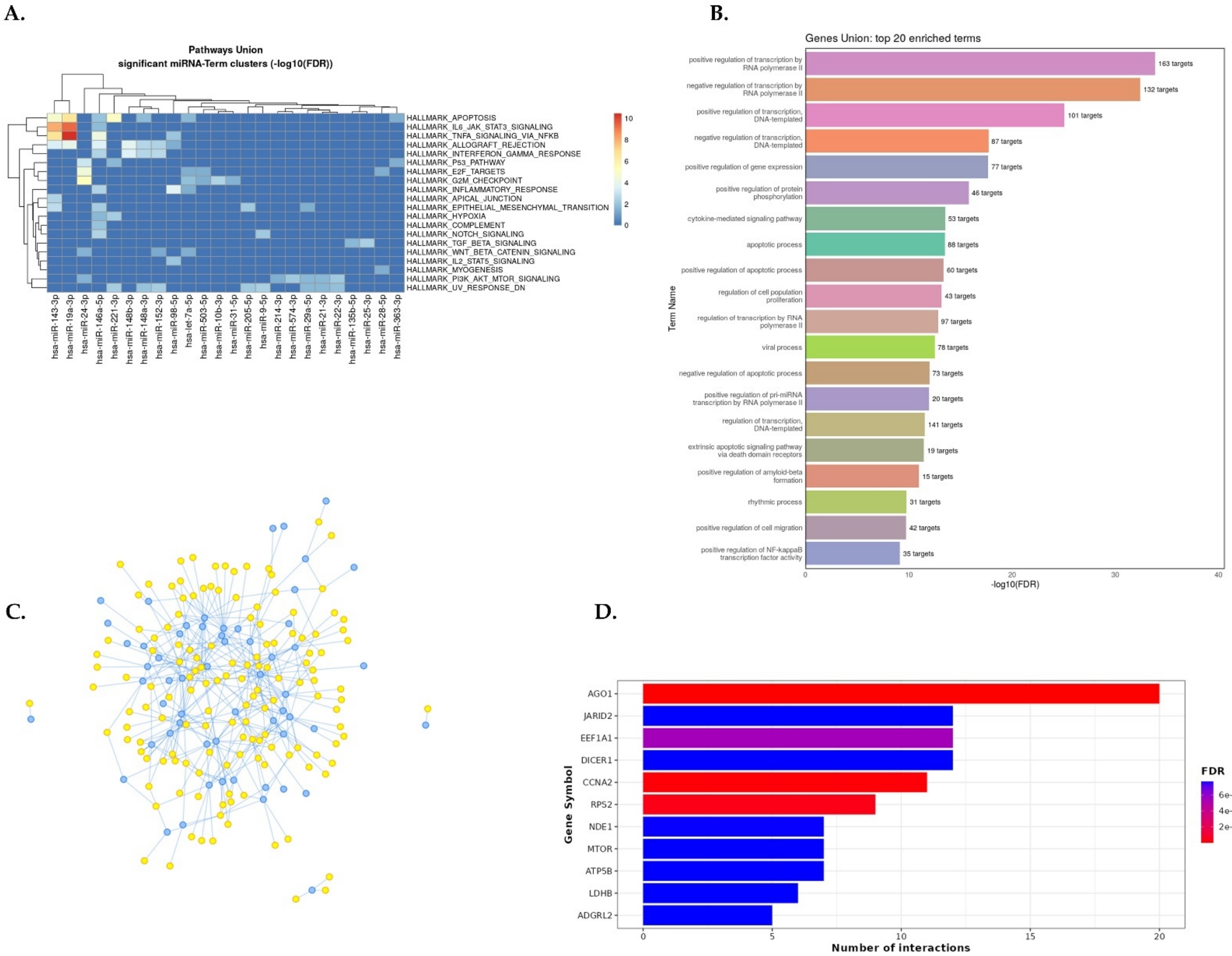

Footnote. Summary of gene set enrichment analysis of dysregulated miRNAs in MN (miRNA-MN). A. DIANA-miRPath v4.0 analysis for the pathway union of MSigDB hallmark gene sets of significantly dysregulated miRNA signatures. MSigDB pathway union represents well-defined biological states or processes from MSigDB 2023.2 release. **B.** The most strongly enriched 20 GO biological proceses related to miRNA-DN from the MIENTURNET web tool. **C.** Interaction network between miRNAs and target genes; blue dots represent miRNAs, and yellow dots represent target genes. **D**. Bar plot with the top 10 target genes on the Y-axis and the number of miRNAs targeting them are shown on the X-axis. The plot is color-coded by increasing the FDR value from red to blue. Abbreviation: MN: Membranous nephropathy.

Supplementary Figure S14. Summary of gene set enrichment analysis of dysregulated miRNAs in murine model of DKD

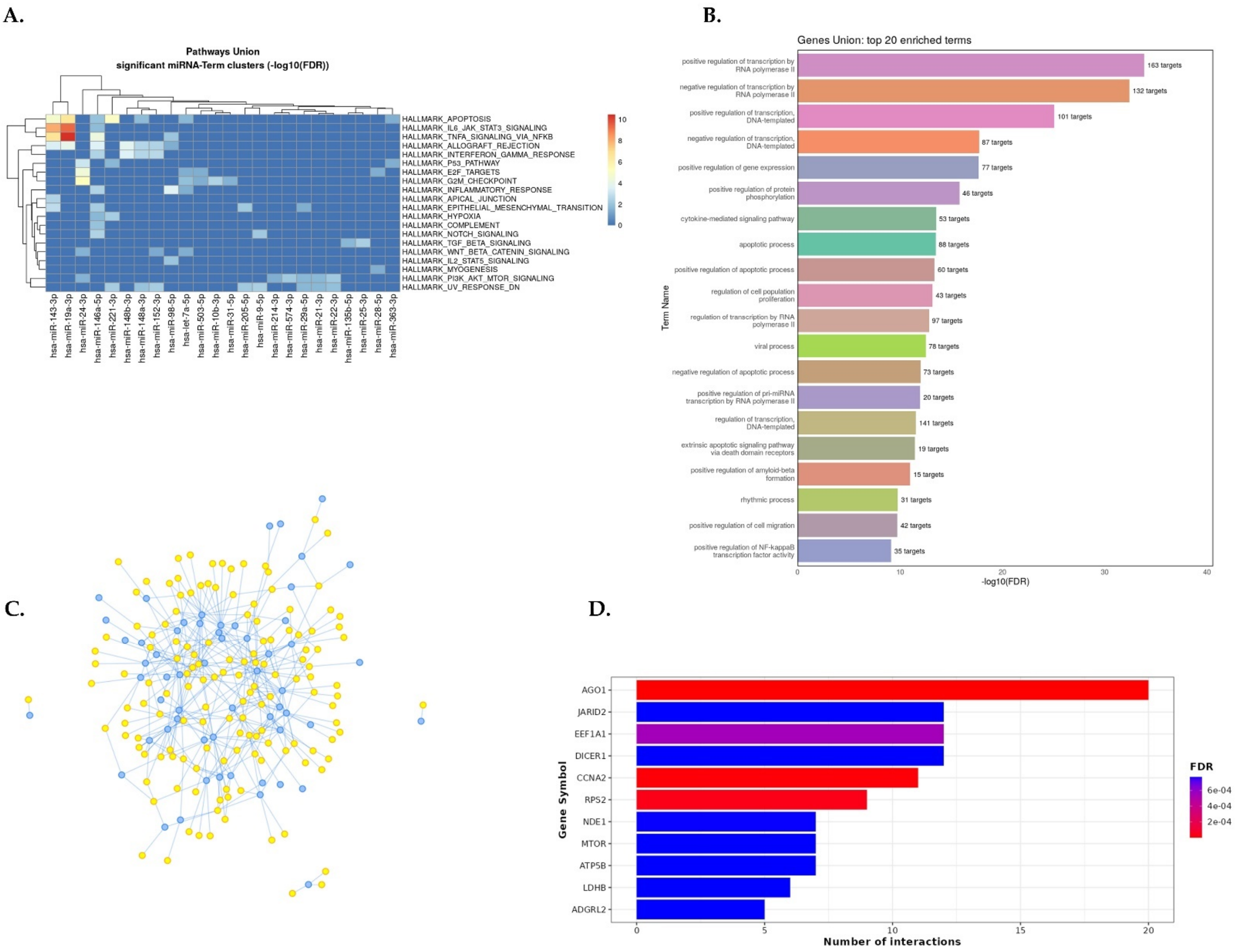

Footnote. Summary of gene set enrichment analysis of dysregulated miRNAs in Murine model of DKD (miRNA-murine DKD). A. DIANA-miRPath v4.0 analysis for the pathway union of MSigDB hallmark gene sets of significantly dysregulated miRNA signatures. MSigDB pathway union represents well-defined biological states or processes from MSigDB 2023.2 release. **B.** The most strongly enriched 20 GO biological proceses related to miRNA-murine DKD from the MIENTURNET web tool. **C.** Interaction network between miRNAs and target genes; blue dots represent miRNAs, and yellow dots represent target genes. **D**. Bar plot with the top 10 target genes on the Y-axis and the number of miRNAs targeting them are shown on the X-axis. The plot is color-coded by increasing the FDR value from red to blue. Abbreviation: DKD: Murine DKD.

Supplementary Table S24. Risk of Bias assessment – MIAME and MIQE tools results for all species

	Study (First author, year)	Risk of bias					
		Raw data	Actual data processing	Sample annotation and experiment variables	Experiment design	Annotation of array design	Experimental data processing protocol
Human studies	A. Ramezani, 2015	I	S	S	S	S	I
	A. Flores-Chova, 2023	I	S	S	S	S	S
	A. Tripathy, 2023	I	S	S	S	S	S
	B. Y. Xu, 2020	S	S	S	S	S	I
	B. Zapała, 2023	I	S	S	S	S	S
	C. Barbagallo, 2019	I	S	S	S	S	S
	C. C. Szeto, 2019	I	S	S	S	S	S
	C.Beltrami, 2018	S	S	S	S	S	S
	D. Delić, 2016	S	S	S	S	S	S
	E. Krasoudaki, 2016	I	S	S	S	S	S
	E. Navarro-Quiroz, 2016	S	S	S	S	S	I
	F. Conserva, 2019	S	S	S	S	S	S
	F.He, 2014	S	S	S	S	S	S
	G. Serino, 2012	S	S	S	S	S	I
	H. Kim, 2019	I	S	S	S	S	S
	I. O. Sun, 2022	I	S	S	S	S	S
	J. D. Massaro, 2019	S	S	S	S	S	I
	J. Yu, 2019	S	S	S	S	S	S
	J. Zhang, 2020	S	S	S	S	S	I
	J. Wu, 2018	S	S	S	S	S	S
	M. A. Baker, 2017	I	S	S	S	S	S
	M. Cardenas-Gonzalez, 2017	I	S	S	S	S	S
	M. Ulbing, 2016	S	S	S	S	S	I
	N. Wang, 2015	S	S	S	S	S	S
	P. Costa-Reis, 2015	S	S	S	S	S	S
	P. Nandakumar, 2017	I	S	S	S	S	S
	Q. H. Min, 2018	I	S	S	S	S	I
	R. Khurana, 2017	I	S	S	S	S	I
	R.Dai, 2023	S	S	S	S	S	S
	T.Konta, 2014	S	S	S	S	S	I
	W. Chen, 2014	I	S	S	S	S	I

	W. Wang, 2015	S	S	S	S	S	S
	W. Zhang, 2014	I	S	S	S	S	S
	X. Liu, 2020	I	S	S	S	S	S
	Y. Dai, 2009	I	S	S	S	S	S
	Z. Wang, 2020	S	S	S	S	S	S
	Y.Pan, 2018	S	S	S	S	S	S
	Z.Gao, 2020	S	S	S	S	S	S
Murine studies	A.C. Chung, 2010	S	S	S	S	S	S
	B. N. Chau, 2012	S	S	S	S	S	S
	F. Glowacki, 2013	S	S	S	S	S	S
	G. Du, 2017	S	S	S	S	S	S
	H. Ishii, 2021	S	S	S	S	S	S
	J. Long, 2010	S	S	S	S	S	S
	K. Yanai, 2020	S	S	S	S	S	S
	R. Bijkerk, 2016	S	S	S	S	S	S
	R. Morizane, 2014	S	S	S	S	S	S
	X. Zhu, 2016	S	S	S	S	S	S
	Y. Zhang, 2015	S	S	S	S	S	S
	Z. Zhang, 2009	S	S	S	S	S	S
S – sufficient, I –insufficient, n/a –not applicable							

MIAME and MIQE tools - definitions of domains

The six most critical elements contributing to MIAME are:

1. **Raw data**

Sufficient: A raw data provided by CEL or FASTQ file format for CKD and healthy control groups.

Insufficient: An incomplete data provided by CEL or FASTQ file format for CKD and healthy control groups.

Not reported: Not reported.

2. **Actual data processing.**

Sufficient: A final processed (normalized) data provided for each study group

Insufficient: Data normalization not conducted or insufficient information of data processing.

Not reported: Not reported.

3. **Sample annotation and experiment variables**

Sufficient: A clear definition of sample annotation including tissue, sex and age and the experimental factors and their values (e.g., compound and dose in a dose response study).

Insufficient: An incomplete definition of sample annotation including tissue, sex and age and the experimental factors and their values (e.g., compound and dose in a dose response study).

Not reported: Not reported.

4. **Experiment design**

Sufficient: A clear definition of experiment design including which raw data file relates to CKD and healthy control groups, and technical and biological replicates reported.

Insufficient: An incomplete definition of experiment design including which raw data file relates to CKD and healthy control groups, and technical and biological replicates reported.

Not reported: Not reported.

5. **Annotation of array design**

Sufficient: A clear definition of annotation of the array or sequence features examines including gene identifiers and genomic coordinates.

Insufficient: An incomplete definition of annotation of the array or sequence features examines including gene identifiers and genomic coordinates.

Not reported: Not reported.

6. **Experimental data processing protocol**

Sufficient: A clear definition of what normalization method has been used to obtain the final processed data.

Insufficient: An incomplete definition of what normalization method has been used to obtain the final processed data.

Not reported: Not reported.

Supplementary Table S25. Risk of Bias assessment - SYRCLE's RoB tool results for murine studies

	Risk of bias										Rating
Study (First author, year)	Random sequence allocation	Baseline characteristics	Concealment of allocation	Random housing	Blinding of interventions	Randomization of outcome assessment	Blinding of outcome assessment	Reporting of missing data	Selective outcome reporting	Other	**Overall risk of bias**
A.C. Chung, 2010	☺	☺	☺	☺	n/a	☺	n/a	☺	😐	☺	😐
B. N. Chau, 2012	☺	☺	☺	☺	n/a	☺	n/a	☺	😐	☺	😐
F. Glowacki, 2013	☺	☺	☺	☺	n/a	☺	n/a	☺	😐	☺	😐
G. Du, 2017	☺	☺	☺	☺	n/a	☺	n/a	☺	😐	☺	😐
H. Ishii, 2021	☺	☺	☺	☺	n/a	☺	n/a	☺	☺	☺	☺
J. Long, 2010	☺	☺	☺	☺	n/a	☺	n/a	☺	☺	☺	☺
K. Yanai, 2020	☺	☺	☺	☺	n/a	☺	n/a	☺	☺	☺	☺
R. Bijkerk, 2016	☺	☺	☺	☺	n/a	☺	n/a	☺	☺	☺	☺
R. Morizane, 2014	☺	☺	☺	☺	n/a	☺	n/a	☺	😐	☺	😐
X. Zhu, 2016	☺	☺	☺	☺	n/a	☺	n/a	☺	😐	☺	😐
Y. Zhang, 2015	☺	☺	☺	☺	n/a	☺	n/a	☺	😐	☺	😐
Z. Zhang, 2009	☺	☺	☺	☺	n/a	☺	n/a	☺	☺	☺	☺
☺ : low risk; 😐 : unclear, n/a: not applicable											

SYRCLE's RoB_tool - definitions of domains

1. **Selection bias: Random sequence allocation**

Low: A clear description of the method and allocation sequence adequately generated and applied.

Unclear: An incomplete description of method, and allocation sequence generation and application are not clear.

High: A missing description of method, and allocation sequence not adequately generated and applied.

2. **Selection bias: Baseline characteristics**

Low: A clear description of the possible prognostic factors or animal characteristics, study groups were similar at the baseline or they adjusted for confounders in the analysis.

Unclear: An incomplete description of the possible prognostic factors or animal characteristics, baseline characteristic among study groups are unclear.

High: A missing description of possible prognostic factors or animal characteristics, baseline characteristic among study groups not defined.

3. **Selection bias: Concealment of allocation**

Low: A clear description of the method used to conceal the allocation sequence and allocation adequately concealed.

Unclear: An incomplete description of the method used to conceal the allocation sequence and allocation concealment is not clear.

High: A missing description of the method used conceals the allocation sequence and allocation concealment is not mentioned.

4. **Performance bias: Random housing**

Low: A clear description of all measures used, animals randomly housed during the experiment.

Unclear: An incomplete description of all measures used, randomization of animal house is not clear.

High: A missing description of all measures used, animals are not randomly housed during the experiment.

5. **Performance bias: Blinding of interventions**

Low: A clear description of all measures used, caregivers and/or investigators blinded from knowledge which intervention each animal received during the experiment.

Unclear: An incomplete description of all measures used, blinding process of caregivers and/or investigators from knowledge which intervention each animal received during the experiment is not clear.

High: A missing description of all measures used, caregivers and/or investigators are not blinded from knowledge which intervention each animal received during the experiment.

6. **Detection bias -Randomization of outcome assessment**

Low: A clear description of animal selection method, animals were selected at random for outcome assessment.

Unclear: An incomplete description of the animal selection method, animal selection for outcome assessment is unclear.

High: A missing description of animal selection method, animals were not selected at random for outcome assessment.

7. **Detection bias: Blinding of outcome assessment**

Low: A clear description of all measures used and effective intended blinding procedure including outcome assessor.

Unclear: An incomplete description of all measures used and effective intended blinding procedure including outcome assessor.

High: A missing description of all measures used and effective intended blinding procedure including outcome assessor.

8. **Attrition bias: Reporting of missing data**

Low: A clear description of completeness of outcome data for each main outcome, including attrition and exclusion from the analysis. If any, incomplete outcome data adequately addressed.

Unclear: An incomplete description of completeness of outcome data for each main outcome, including attrition and exclusion from the analysis. If any, address of incomplete outcome data is not clear.

High: A missing description of completeness of outcome data for each main outcome, including attrition and exclusion from the analysis. If any, incomplete outcome data not adequately addressed.

9. **Reporting bias: Selective outcome reporting**

Low: All results of the study free of selective outcome reporting.

Unclear: The outcome reporting is not clear.

High: The selective outcome reporting is applied.

10. **Other bias** - Other

Low: Study apparently free of other problems that could results in high risk of bias (for example, experimental unit).

Unclear: Study status from other problems that could results in high risk of bias is not clear.

High: Study is not free of other problems that could results in high risk of bias (for example, using experimental unit for each animal as a cage unit or reverse).

Supplementary Figure S15A. Risk of Bias assessment – SYRCLE tool results

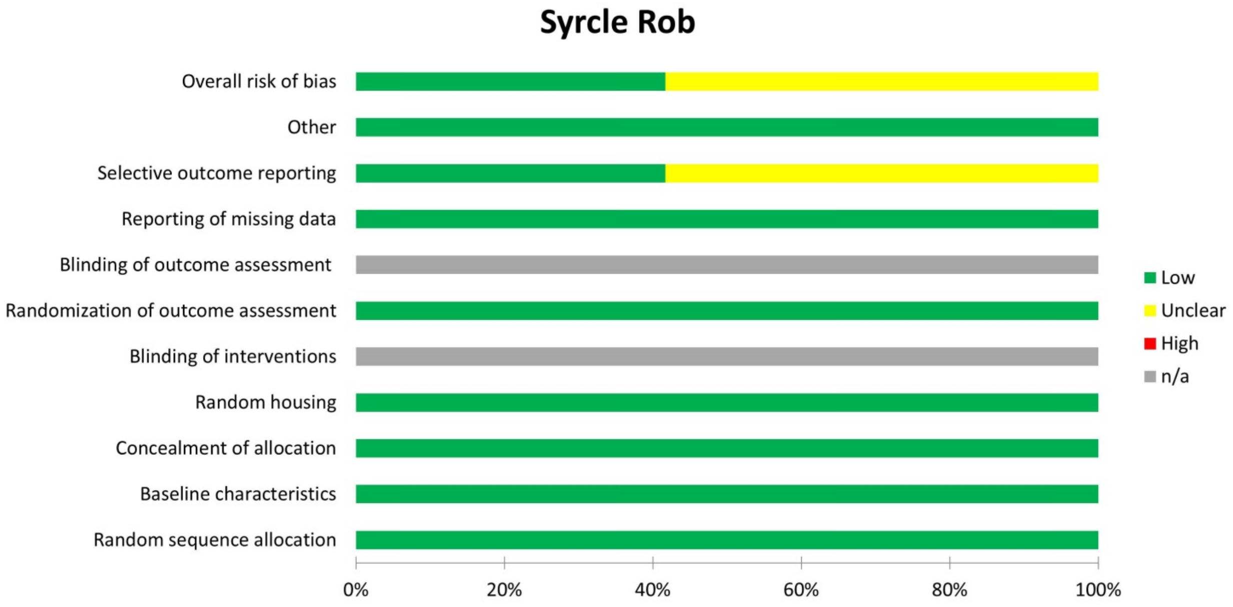

Footnote: Quality assessment according to the SYRCLE guideline. Green bars, yellow bars, and grey bars, respectively, indicate the items that were sufficient in annotation, unclear in annotation, and not reported.

Supplementary Figure S15B. Risk of Bias assessment – MIAME and MIQE tools results for all species.

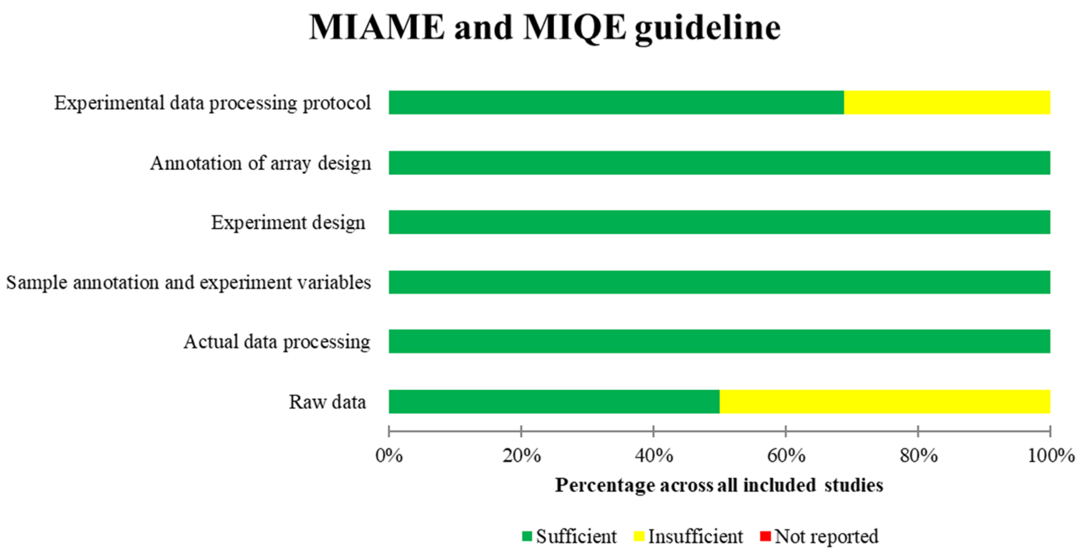

Footnote: Quality assessment according to the MIAME and MIQE guidelines. Green bars, yellow bars, and grey bars, respectively, indicate the items that were sufficient in annotation, not sufficient in annotation, and not reported.

Supplementary Table S26. PRISMA 2020 checklist

Section and Topic	Item #	Checklist item	Location where item is reported
**TITLE**			
Title	1	Identify the report as a systematic review.	Page 1
**ABSTRACT**			
Abstract	2	See the PRISMA 2020 for Abstracts checklist.	Page 1
**INTRODUCTION**			
Rationale	3	Describe the rationale for the review in the context of existing knowledge.	Page 2-3
Objectives	4	Provide an explicit statement of the objective(s) or question(s) the review addresses.	Page 2-3
**METHODS**			
Eligibility criteria	5	Specify the inclusion and exclusion criteria for the review and how studies were grouped for the syntheses.	Page 3, Supplementary method
Information sources	6	Specify all databases, registers, websites, organisations, reference lists and other sources searched or consulted to identify studies. Specify the date when each source was last searched or consulted.	Page 3, Supplementary method
Search strategy	7	Present the full search strategies for all databases, registers and websites, including any filters and limits used.	Page 4-5, Supplementary method
Selection process	8	Specify the methods used to decide whether a study met the inclusion criteria of the review, including how many reviewers screened each record and each report retrieved, whether they worked independently, and if applicable, details of automation tools used in the process.	Page 4-5, Supplementary method
Data collection process	9	Specify the methods used to collect data from reports, including how many reviewers collected data from each report, whether they worked independently, any processes for obtaining or confirming data from study investigators, and if applicable, details of automation tools used in the process.	Page 4-5, Supplementary method
Data items	10a	List and define all outcomes for which data were sought. Specify whether all results that were compatible with each outcome domain in each study were sought (e.g. for all measures, time points, analyses), and if not, the methods used to decide which results to collect.	Page 4-5, Supplementary method
	10b	List and define all other variables for which data were sought (e.g. participant and intervention characteristics, funding sources). Describe any assumptions made about any missing or unclear information.	Page 4, Supplementary method
Study risk of bias assessment	11	Specify the methods used to assess risk of bias in the included studies, including details of the tool(s) used, how many reviewers assessed each study and whether they worked independently, and if applicable, details of automation tools used in the process.	Page 16
Effect measures	12	Specify for each outcome the effect measure(s) (e.g. risk ratio, mean difference) used in the synthesis or presentation of results.	Page 15
Synthesis methods	13a	Describe the processes used to decide which studies were eligible for each synthesis (e.g. tabulating the study intervention characteristics and comparing against the planned groups for each synthesis (item #5)).	Page 16
	13b	Describe any methods required to prepare the data for presentation or synthesis, such as handling of missing summary statistics, or data conversions.	Page 4, Supplementary method
	13c	Describe any methods used to tabulate or visually display results of individual studies and syntheses.	Page 15
	13d	Describe any methods used to synthesize results and provide a rationale for the choice(s). If meta-analysis was performed, describe the model(s), method(s) to identify the presence and extent of statistical heterogeneity, and	Page 15

Section and Topic	Item #	Checklist item	Location where item is reported
		software package(s) used.	
	13e	Describe any methods used to explore possible causes of heterogeneity among study results (e.g. subgroup analysis, meta-regression).	Page 4-5, Supplementary method
	13f	Describe any sensitivity analyses conducted to assess robustness of the synthesized results.	Page 4-5, Supplementary method
Reporting bias assessment	14	Describe any methods used to assess risk of bias due to missing results in a synthesis (arising from reporting biases).	Page 4-5, Supplementary method
Certainty assessment	15	Describe any methods used to assess certainty (or confidence) in the body of evidence for an outcome.	NA
**RESULTS**			
Study selection	16a	Describe the results of the search and selection process, from the number of records identified in the search to the number of studies included in the review, ideally using a flow diagram.	Page 2
	16b	Cite studies that might appear to meet the inclusion criteria, but which were excluded, and explain why they were excluded.	Page 3, Supplementary file 1-3
Study characteristics	17	Cite each included study and present its characteristics.	Table 1 and 2
Risk of bias in studies	18	Present assessments of risk of bias for each included study.	Table S. 24-25, Figure S15A and B
Results of individual studies	19	For all outcomes, present, for each study: (a) summary statistics for each group (where appropriate) and (b) an effect estimate and its precision (e.g. confidence/credible interval), ideally using structured tables or plots.	Figure 2-3<br>Figure S1-14
Results of syntheses	20a	For each synthesis, briefly summarise the characteristics and risk of bias among contributing studies.	Figure S 15A-B
	20b	Present results of all statistical syntheses conducted. If meta-analysis was done, present for each the summary estimate and its precision (e.g. confidence/credible interval) and measures of statistical heterogeneity. If comparing groups, describe the direction of the effect.	Table S. 24-25, Figure S15A and B
	20c	Present results of all investigations of possible causes of heterogeneity among study results.	Table S. 24-25, Figure S15A and B
	20d	Present results of all sensitivity analyses conducted to assess the robustness of the synthesized results.	Supplementary file 1, Figure S16
Reporting biases	21	Present assessments of risk of bias due to missing results (arising from reporting biases) for each synthesis assessed.	Table S24-25<br>Figure S15A and B
Certainty of evidence	22	Present assessments of certainty (or confidence) in the body of evidence for each outcome assessed.	NA
**DISCUSSION**			
Discussion	23a	Provide a general interpretation of the results in the context of other evidence.	Page 11
	23b	Discuss any limitations of the evidence included in the review.	Page 15
	23c	Discuss any limitations of the review processes used.	Page 15
	23d	Discuss implications of the results for practice, policy, and future research.	Page 14
**OTHER INFORMATION**			
Registration and protocol	24a	Provide registration information for the review, including register name and registration number, or state that the review was not registered.	Page 15
	24b	Indicate where the review protocol can be accessed, or state that a protocol was not prepared.	Page 15
	24c	Describe and explain any amendments to information provided at registration or in the protocol.	NA

Section and Topic	Item #	Checklist item	Location where item is reported
Support	25	Describe sources of financial or non-financial support for the review, and the role of the funders or sponsors in the review.	Page 17
Competing interests	26	Declare any competing interests of review authors.	Page 17
Availability of data, code and other materials	27	Report which of the following are publicly available and where they can be found: template data collection forms; data extracted from included studies; data used for all analyses; analytic code; any other materials used in the review.	Page 17

From: Page MJ, McKenzie JE, Bossuyt PM, Boutron I, Hoffmann TC, Mulrow CD, et al. The PRISMA 2020 statement: an updated guideline for reporting systematic reviews. BMJ 2021;372:n71. Doi: 10.1136/bmj.n71

For more information, visit: http://www.prisma-statement.org/

Supplementary Figure S16. Validation of miR-936 expression in HK-2 human proximal tubular cell line and in diabetic kidney biopsy samples

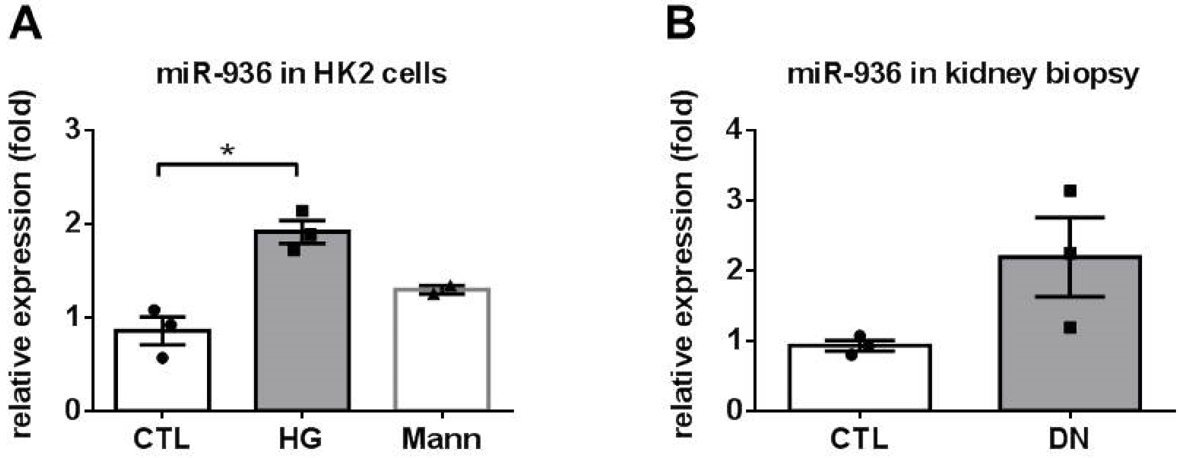

Footnote: A: Twenty-four hour incubation of HK-2 cells in high glucose medium (HG, 25 mmol/l glucose) increased miR-936 expression by 2-fold as compared to control cells (CTL, 5 mmol/l glucose). Mannitol addition (at 20 mmol/l) to normal medium as osmotic control had no effect. n=3/group, * $p<0.05$ by Kruskal Wallis test.
B: Kidney biopsies from diabetic nephropathy patients (DN, n=3) tended to have 2.2-fold over-expression of miR-936 as compared to control kidney tissues (CTL, n=3), yet did not reach statistical significance (Mann-Whitney test).

Supplementary Figure S17. Subgroup analysis of platform in DN.

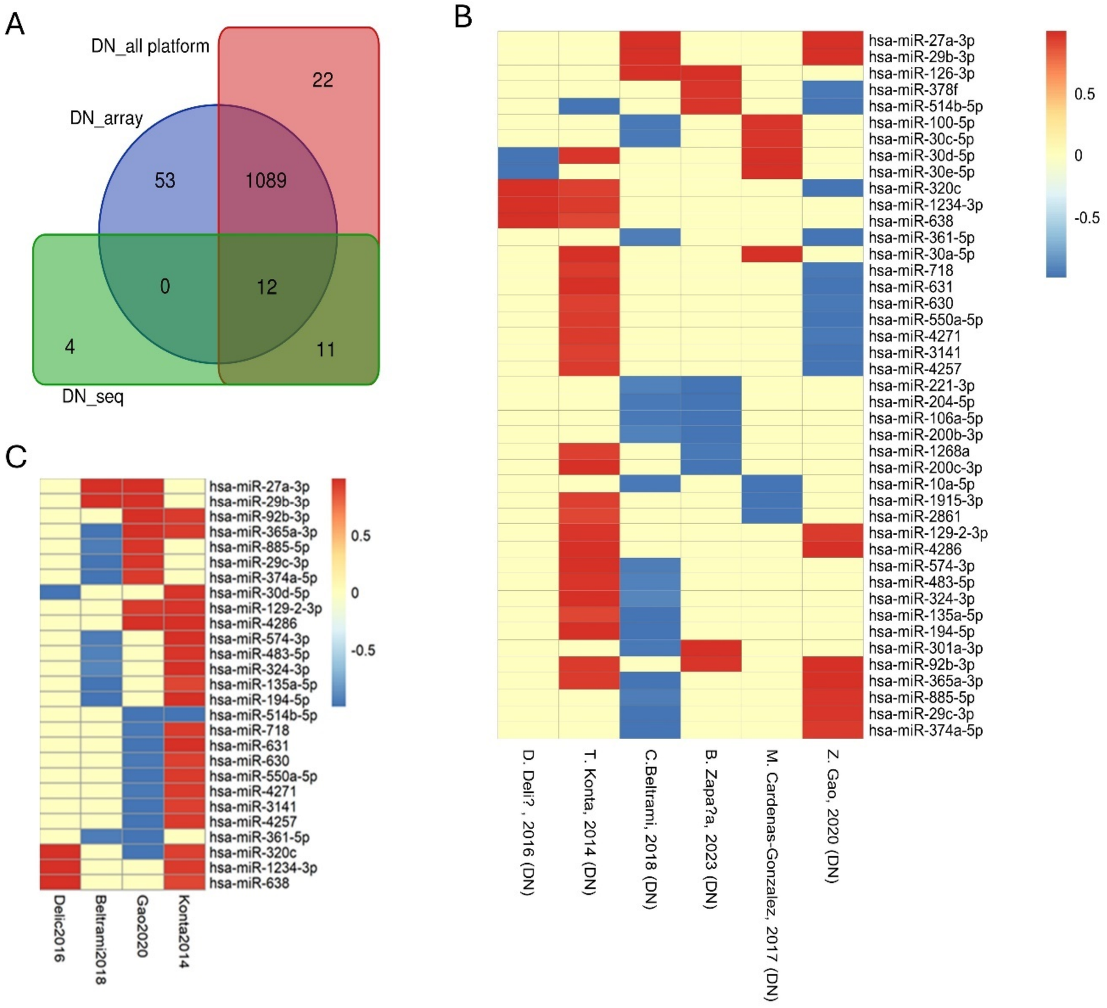

Footnote: A. The Venn diagram compares total dysregulated miRNAs in DN by subgroups of technical platforms. Fifty-three miRNAs are differentially expressed in the microarray subgroup compared to the combination of microarray and next-generation sequencing. Four miRNAs are differentially expressed in the next-generation sequencing subgroup compared to microarray or combination. **B** and **C**. The heat map illustrates the dysregulated urinary miRNAs of DN patients compared to controls according to the heat map of the six (B) and four (C) identified eligible studies using an average ranking score. B represents miRNAs tested by all type of platforms. C represents miRNAs tested by only MicroArray. Results represent only miRNAs tested by Microarray. The listed miRNAs were reported in at least two expression profiling studies. Every row corresponds to an individual miRNA, and each column indicates an individual study. The three different colors represent the presence and dysregulation direction of individual miRNAs ranging from 1 to -1 as follows: absent (0 -yellow), up-regulated (0.1 to 1 -red), and downregulated (-0.1 to -1 -blue). If the opposite direction of the individual miR exists, it is provided as blue or red in the corresponding row. Abbreviation: DN_all platform: miRNAs tested by all available technical platforms; DN_seq: miRNAs tested by only next-generation sequencing (data is only available in blood samples); DN_array: miRNAs tested by only microarray (data is only available in urine and kidney tissue samples).
